# Molecular changes during AT/RT progression associated with epithelial–mesenchymal transition and extracellular matrix changes

**DOI:** 10.1007/s00401-026-03050-1

**Published:** 2026-07-01

**Authors:** Lea Altendorf, Anton Althammer, Rajanya Roy, Karoline Hack, Flavia W. de Faria, Arend Koch, Vanessa Thaden, Melanie Schoof, Martin U. Schuhmann, Peter Hauser, Pascal D. Johann, Martin Hasselblatt, Michael C. Frühwald, Kornelius Kerl, Ulrich Schüller

**Affiliations:** 1 Department of Pediatric Hematology and Oncology, University Medical Center Hamburg-Eppendorf, 20251 Hamburg, Germany; 2 Research Institute Children’s Cancer Center Hamburg, Martinistraße 52, N63, 20251 Hamburg, Germany; 3 Mildred Scheel Cancer Career Center HaTriCS4, University Medical Center Hamburg-Eppendorf, 20251 Hamburg, Germany; 4 Department of Pediatric Hematology and Oncology, University Medical Center Münster, 48149 Münster, Germany; 5 Institute of Neuropathology, Charité – Universitätsmedizin Berlin, 10117 Berlin, Germany; 6 Division of Pediatric Neurosurgery, Department of Neurosurgery, Eberhard Karl’s University Hospital of Tübingen, 72076 Tübingen, Germany; 7 Second Department of Pediatrics, Semmelweis University, 1085 Budapest, Hungary; 8 Paediatric and Adolescent Medicine, Swabian Children’s Cancer Center Augsburg, EU-RHAB Trial Center, 86156 Augsburg, Germany; 9 Hopp Children’s Cancer Center (KiTZ), German Cancer Research Center (DKFZ) and German Cancer Research Consortium (DKTK), 69120 Heidelberg, Germany; 10 Division of Pediatric Neurooncology, German Cancer Research Center (DKFZ) and German Cancer Research Consortium (DKTK), 69120 Heidelberg, Germany; 11 Institute of Neuropathology, University Hospital Münster, 48149 Münster, Germany; 12 Institute of Neuropathology, University Medical Center Hamburg-Eppendorf, 20251 Hamburg, Germany

**Keywords:** AT/RT, Tumor recurrence, Therapy resistance, Single-nucleus RNA sequencing, pEMT

## Abstract

Atypical teratoid/rhabdoid tumors (AT/RT) are the most common malignant brain tumors during infancy and associated with a dismal prognosis. The majority of patients suffer from tumor progression or recurrence, but underlying mechanisms remain unknown. To better understand such mechanisms, we performed single-nucleus RNA sequencing (snRNAseq) of eight paired primary tumors and recurrences. Tumor cells and cells of the tumor microenvironment (TME) were analyzed separately. Potentially therapy-resistant tumor cells were identified through the comparison of global gene expression profiles between primary and recurrent tumor cell populations using *CIBERSORT*. Histopathology, in vitro experiments, bulk RNA sequencing, and survival analysis were performed for validation. Paired primary and recurrent AT/RT showed significant differences in their gene expression profiles. Potentially therapy-resistant AT/RT-MYC tumor cells revealed changes in the extracellular matrix (ECM) as well as altered developmental processes and immune signaling pathways. Respective gene signatures were correlated with inferior survival in AT/RT-MYC patients. Tumor cells of relapsed AT/RT-MYC underwent partial epithelial–mesenchymal transition (pEMT), a feature that was confirmed by immunohistochemistry (IHC) and by analyzing AT/RT cells after standard therapy in vitro. Together, we identified potential mechanisms of tumor relapse and therapy resistance in AT/RT, which could be employed to improve therapy in future.

**Supplementary Information:**

The online version contains supplementary material available at 10.1007/s00401-026-03050-1.

## Introduction

AT/RT are malignant tumors of the central nervous system (CNS) mainly affecting young children. They are characterized by an aggressive, infiltrative growth and a very poor prognosis, with a median overall survival (OS) ranging from 9 to 17 months, depending on risk groups [17].

AT/RT are assigned to four different DNA methylation subtypes. These differ regarding their clinical characteristics, genetics, and gene expression profiles [25, 30]. 95% of all AT/RT patients belong to the three subtypes AT/RT-SHH, AT/RT-MYC, and AT/RT-TYR that are characterized by *SMARCB1* mutations [7, 28]. In very rare cases, a *SMARCA4* mutation is the underlying genetic cause [23, 50]. These cases belong to a distinct subtype, AT/RT-SMARCA4 [25]. Both SMARCB1 and SMARCA4 are part of the SWItch/Sucrose Non-Fermentable (SWI/SNF) complex, which is involved in the packaging of the DNA to histones and therefore, the accessibility of the DNA and the regulation of gene expression. They are also known to act as tumor suppressors [60]. Often, the mutations are already present in the germline, which is associated with the rhabdoid tumor predisposition syndrome 1 (RTPS1; OMIM #609322) for patients with *SMARCB1* mutations and rhabdoid tumor predisposition syndrome 2 (RTPS2; OMIM #613325) for *SMARCA4*-mutated patients. AT/RT patients received surgery followed by chemotherapy. Radiotherapy is not generally recommended for patients younger than 3 years but is applied on a single case basis, and targeted therapies are still lacking. In line with the fact that the prognosis is very poor, AT/RT often progress or recur, matching the observation of pronounced therapy-resistance in AT/RT patients [18]. Previously, differences between paired primary and recurrent AT/RT, regarding their histology, proliferative activity, copy number and bulk gene expression profiles were identified [29]. Nevertheless, the identification of therapy-resistant tumor cells is outstanding. Thorough characterization of therapy-resistant and recurrent AT/RT tumor cells is urgently needed to identify novel therapeutic targets overcoming resistance.

Here, we describe the transcriptomic differences between eight paired AT/RT primary and recurrent tumors on a single-nucleus level. Furthermore, primary tumor cells with similarity to paired recurrences were identified and characterized in this study. We assume that such cells are therapy-resistant and develop to recurrences. Characterization of these cells and respective mechanisms might therefore result in the identification of novel treatment targets. In this framework, we identified increased ECM remodeling and pEMT in the context of therapy resistance.

## Methods

### Data sets

Patient data were derived from the databases of the EU-RHAB registry. EU-RHAB has received continuous ethical approval (Ethics Committee University of Münster, Germany 2009-532-f-S, most recently amended 17AUG2021). Case numbers 1–26 were assigned to the patients according to Johann et al. [28]. Cases 27 and 28 were added to this study. Clinical and molecular information is summarized in Fig. S1a and Table S1.

### Isolation of cells from FFPE tissue and snRNAseq

snRNAseq of formalin-fixed paraffin-embedded (FFPE) tissue was performed with the Chromium Single Cell Flex Gene Expression Assay (10× Genomics). Cells were isolated according to the demonstrated protocol “Isolation of Cells from FFPE Tissue Sections for Chromium Fixed RNA Profiling”, Document Number CG000632 Rev B (10× Genomics) using a gentleMACS Octo Dissociator (#130-134-029, Miltenyi Biotec). FFPE scrolls were dissociated using the Enzyme Mix containing Liberase TH (# 5401151001, Millipore Sigma). Probe hybridization and multiplex library preparation were performed using the 10× Genomics Sample Preparation Kit PN-1000414, 16 rxns, according to the protocol “Chromium Fixed RNA Profiling Reagent Kits for Multiplexed Samples”, Document Number CG000527 Rev E (10× Genomics). The resulting pooled libraries, with one barcode per tumor sample, were sequenced as dual-index libraries in the sequencing core facility of the University Medical Center Münster, on NextSeq 2000 and NovaSeq 6000 (Illumina) sequencing systems, and a recovery of 80,000 cells for each library was targeted.

### snRNAseq data processing, integration, and clustering

The *10*× *Genomics Cell Ranger v8.0.1* software (RRID:SCR_017344) was used for demultiplexing, barcode processing and assignment, single-nucleus counting, alignment to the human reference genome GRCh38-2024-A, quality control, and finally for Feature Barcode Matrix generation. Filtered Feature Barcode Matrices for each sample were processed in *R Studio* (version 4.4.1). Normalization, scaling, principal component analysis (PCA), shared nearest neighbor graph construction (snn graph), clustering and uniform manifold approximation and projection (UMAP) were performed via the *Seurat* (version 5.1.0, RRID:SCR_007322) pipeline [22]. Data were merged and normalized using the *SCTransform()* function, correcting for the percentage of mitochondrial counts. Only nuclei with more than 200 and < 2500 detected genes and < 5% mitochondrial reads were selected. Processed data were visualized in UMAPs using 10 neighboring points in local approximation of manifold structure.

A *CCA* integration using the *FindIntegrationAnchors()* and *IntegrateData()* functions, both included in the *Seurat* package, was performed to identify resistant primary tumor cells over all samples, as well as for integration of AT/RT-MYC primary tumors that did not relapse.

TME cells were extracted from the raw data based on gene expression profiles and copy-number variations (CNVs). CNVs were investigated using the *inferCNV* package (version 1.22.0, RRID:SCR_021140) [56] with the chromosome information from hg38. TME cells identified by gene expression profiles were used as reference cells and all potential tumor cells as input. CNVs were calculated using *infercnv::run* (settings: analysis_mode = “samples”, cutoff = 0.1, cluster_by_groups = TRUE, denoise = TRUE, HMM = TRUE).

Normalization and processing were done as described above. Integration was performed utilizing *RunHarmony()* from the R package *harmony* (version 1.2.3) [57] with the sample ID as a covariate after *RunPCA()* following the developers’ recommendations. To compare the gene expression profiles between the DNA methylation subtypes, pseudobulk values for each sample were calculated using the *AggregateExpression()* function. The *DESeq2* package (version 1.46.0, RRID:SCR_015687) [36] was used to identify the 1,000 most variable genes between the DNA methylation subtypes. A Consensus clustering was performed using the *ConsensusClusterPlus* package (version 1.70.0, RRID:SCR_016954) [37, 60] and the distance was defined as *“pearson”*. The optimal *k* was calculated as *k* = 3. To determine whether primary tumor and recurrence of one patient have distinct gene expression profiles, pseudobulk values were calculated as described above. Pseudobulk values were normalized using *VST normalization*, included in the *DESeq2* package. The transcriptomic divergence was calculated as 1 minus the *Pearson* correlation between pseudobulk profiles of paired primary and recurrent tumors. A divergence of 0 means that primary tumor and paired recurrence have very similar transcriptomes and a divergence of 1 indicates distinct transcriptomes. Results were visualized in a lollipop plot created with *ggplot2* (version 3.5.1, RRID:SCR_014601) [58]. Statistics was perfomed using a two-sided Wilcoxon test.

### Annotation of the tumor microenvironment (TME)

The TME was annotated in a two-step procedure of automated and manual cross-labeling as recommended [24]. Automated annotation was performed utilizing the *Azimuth* (version 0.5.0, RRID:SCR_021084) algorithm [21] following the developers’ recommendations. Therefore, *RunAzimuth()* was called with the herein published TME as query dataset and a custom reference built via *AzimuthReference()* from a high-quality TME dataset of eleven glioblastomas previously published as reference dataset [42]. Manual annotation was performed based on canonical markers. For myelomonocytic cells (monocytes, macrophages, and DCs), an additional published meta-analysis was used to verify annotation [39].

### Differential abundance testing for the TME

Differential abundance testing was performed by assigning cells to partially overlapping neighborhoods on a nearest neighbor graph and subsequently fitting a generalized linear model (GLM) to account for the developmental nature of especially myelomonocytic cells. Therefore, we utilized the R package *miloR* (version 2.3.1, RRID:SCR_025630) [12]. A nearest neighbor graph was imported from *Seurat’s FindNeighbors()* function via *miloR’s buildFromAdjacency()* function, as described in the developers’ vignettes. Grouping into overlapping neighborhood groups, visualization, and statistical testing followed the developers’ recommendations. For the latter, a GLM comparing primary versus recurrent tumors was fitted, incorporating tumor DNA methylation subtype, patient ID, recurrence status, and experimental batches as covariates. Correction for multiple testing was performed by calculating the spatial false discovery rate (sFDR < 0.1) accounting for overlap between neighborhoods following the developers’ recommendations. Neighborhoods were mapped back to the original populations to identify the differentially abundant populations.

### Cell–cell communication

To identify potential interactions between cells with consecutive activation of downstream gene targets, we utilized the package *nichenetr* (version 2.2.0) [10] following the developers’ recommendations. Myelomonocytic cells were set as receivers, whereas tumor cells and all TME cells were set as sender cells. An expression of 5% of one population’s cells was used as the minimum threshold to include ligand-target pairs in the analysis. The top ten activated genes were selected to perform ligand-target predictions.

### CIBERSORT analysis

As the basis for the *CIBERSORT* analysis (RRID:SCR_016955), integrated data sets of all AT/RT-MYC primary tumor cells and all AT/RT-MYC recurrence tumor cells, with a resolution of 0.5 for *Seurat* cluster assignment (adequate resolution for snRNAseq data sets of around 3000 cells) [21, 22], were used. Pseudobulk values for each *Seurat* cluster were calculated using the *AggregateExpression()* function, and similarities between the single clusters were estimated using *CIBERSORT()* function (perm = 500) of the *IOBR* package (version 0.99.0, RRID:SCR_025619) [40].

*CIBERSORT* was also used for the deconvolution of previously published bulk RNA sequencing (bulk RNAseq) data [29]. The herein published snRNAseq data served as a reference with tumor cells grouped by their respective DNA methylation subtype and TME cells by their neighborhood group calculated via *miloR* (version 2.3.1, RRID:SCR_025630) [12]. Pseudobulk values for each of these groups were calculated. Comparisons between primary and recurrent samples were performed via a one-sided Student’s *t*-test, with *p* < 0.05 being considered to indicate statistical significance. As a validation of this method, a *Pearson* correlation was performed by calculating pseudobulk values for each *Seurat* cluster of the primary tumors and for the recurrences overall. The common genes were chosen and the correlation was calculated using the *cor(method* = *“pearson”)* function of the *stats* package (version 4.4.1, RRID:SCR_025968) [43]. The results were visualized in a heatmap using the *pheatmap* package (version 1.0.12, RRID:SCR_016418) [33].

### Differentially expressed genes (DEG) and pathways

DEG between the different tumor clusters were identified using the *FindAllMarkers()* function of the *Seurat* package (version 5.1.0, RRID:SCR_007322). Significant DEG with an adjusted *p *value < 0.05 (Benjamini–Hochberg (BH) adjustment) and a log_2_ fold change > 1 and < -1 were used for pathway analysis. The *Reactome Pathway* (RRID:SCR_003485) database was used for gene set enrichment analysis using the *gsePathway()* function included in the *ReactomePA* package (version 1.50.0, RRID:SCR_019316) [65]. Results were visualized in a *cnetplot* with the *enrichplot* package (version 1.26.6, RRID:SCR_026996) [64].

DEG between different TME cell types or between primary and recurrent samples within one TME cell type were analyzed as specified below using a GLM. Pseudobulk data were created based on the DNA methylation subtype, patient ID, recurrence status, and identified cell type via *Seurat’s AggregateExpression()* function, and DEG of raw count data were analyzed via the package *DESeq2* (version 1.46.0, RRID:SCR_015687) [36], embedded in *Seurat’s FindMarkers()* function following the developers’ recommendations. Genes with an adjusted *p *value < 0.05 after multiple testing correction via BH adjustment were considered to be significant.

### Annotation of cancer cell states

An NMF analysis [5, 39] was performed to identify gene modules, which were annotated as cancer cell states based on the function of respective genes. For this, the *NMF* package (version 0.27, RRID:SCR_023124) [19] with the *brunet* algorithm was used. Using *nmfEstimateRank()*, we determined the appropriate factorization rank (*k*), the rank with the highest cophenetic coefficient, which resulted in *k* = 4 for both AT/RT-MYC primary tumors and recurrences. Therefore, we defined four modules and the top 40 genes of each module were used to annotate the modules. The databases PanglaoDB (RRID:SCR_022580) [16], Gene Ontology (GO; RRID:SCR_002811) [2, 4], g:Profiler (RRID:SCR_006809) [32, 44], and ShinyGO (version 0.85.1, RRID:SCR_019213) [20] were used for annotation of the modules or so-called cancer cell states. The cell state fractions were visualized in bar plots using the *ggplot2* package (version 3.5.1, RRID:SCR_014601) [58]. Statistics were calculated using two-sided paired *t *tests.

To validate our annotation, we assigned tumor cells to commonly used pan-cancer cell states [5]. The cell states were assigned using *AUCell* (version 1.28.0, RRID:SCR_021327) [1] according to the developers’ recommendations.

### Histology and immunohistochemistry (IHC)

All human AT/RT tumor samples were fixed in 4% formaldehyde. Cell lines were pelletized, washed with 1× PBS, and fixed in 4% formaldehyde. After that, the cells were washed three times with 1× PBS with subsequent embedding in 3% agarose. Fixed tumor samples and agarose-embedded cells were dehydrated, embedded in paraffin, and sectioned at 2 µm. Hematoxylin and Eosin (H&E) staining was performed using standard protocols. IHC was done using a Ventana System (Roche) according to the manufacturers’ specifications. The antibodies mouse anti-EMA/anti-Mucin-1 (RRID:AB_2148557, DAKO #M0613, 1:200), mouse anti-CD1A (RRID:AB_563515, Novocastra #NCL-L-CD1a-235, 1:50), and mouse anti-Langerin (RRID:AB_2889342, Cell Marque™ #12D6, 392M-18 7 mL predilute) were used for epithelial membrane antigen (EMA), CD1A and Langerin (CD207) stainings. All stainings were quantified in *ImageJ* (version 1.54f, Java 1.8.0_322, RRID:SCR_003070) by calculating the positive area of scans of the whole slides, normalized to the total tumor area, according to the macro:

imageTitle=getTitle();//

run("Set Measurements…", "area area_fraction limit display redirect=None decimal=3");

run("8-bit");

setAutoThreshold("Default dark");

//run("Threshold…");

setThreshold(0, x);

setOption("BlackBackground", true);

run("Convert to Mask");

run(“Measure”);

The threshold *x* was defined as *x* = 100 for EMA, *x* = 200 for CD1A, and *x* = 150 for CD207. For input images, color deconvolution for H DAB vectors was performed and Color_2 was selected. Results were visualized in box plots created with *ggplot2* (version 3.5.1, RRID:SCR_014601) [58]. Statistics was performed using one-sided and two-sided paired *t *tests.

### Cell culture

The two patient-derived AT/RT-MYC cell lines BT-16 (RRID:CVCL_M156) and CHLA-266 (RRID:CVCL_M149) were used. Cells were cultured according to manufacturer specifications. To generate therapy-resistant cells, each cell line was treated with 1 µM doxorubicin (doxo). After 24 h, media were changed and 5 µM etoposide (eto) was added. Again, after 24 h, media were renewed and 1 µM vincristine (vin) was added. Most of the cells died after treatment, but after about 35 days, resistant cells grew again. Cells were harvested for IHC and RNA sequencing. For the latter, cell pellets were resuspended in Trizol and RNA was isolated according to standard protocols. To investigate the effect of the *ETS1* inhibitor TK216, cells were additionally treated with 1 µM TK216. Cell viability was assessed using the CellTiter-Glo® Luminescent Cell Viability Assay (Promega, #G7570). Luminescence was measured using a Tecan Reader (Infinite M200, Tecan Group AG) up to 56 days after treatment. Luminescence values were normalized to DMSO-treated cells and log-transformed. Cell viability was visualized in *RStudio* (version 4.4.1) using *ggplot2* (version 3.5.1, RRID:SCR_014601) [58]. Statistical analysis was performed using two-sided paired *t *tests.

### Bulk RNA sequencing

RNA concentration was measured using a Qubit 4 Fluorometer (Thermo Fisher Scientific). The quantity for each sample was adjusted to 10 ng. The library was prepared using the SMART-Seq Stranded kit (Takara Bio) according to the manufacturer’s specifications. Sequencing of the library pool was done on a NovaSeq X Plus (Illumina), using 2 × 101 bp. Subsequently, demultiplexing was performed with *Illumina bcl2fastq* (version 2.20, RRID:SCR_015058) and the adapters were trimmed with *Skewer* (version 0.2.2, RRID:SCR_001151) [27]. The reads were aligned to the human genome hg38 using *STAR* (RRID:SCR_004463) [13]. The transcripts were counted as feature counts, and they were used as input for the raw count table, which was processed in *R Studio* (version 4.4.1). Matching HUGO symbols for the ENSEMBL IDs were added using the *biomaRt* package (version 2.62.0, RRID:SCR_019214) [14]. Repetitively counted genes and genes from sex chromosomes were removed. The *DESeq2* package (version 1.46.0, RRID:SCR_015687) [36] was used to identify all DEG with a *p *value < 0.05 after BH adjustment, which were visualized in Volcano Plots using the *EnhancedVolcano* package (version 1.24.0, RRID:SCR_018931) [8].

### Survival analysis

For survival analysis, only patients with known OS, PFS, and clinical status (dead or alive) were included. These data were gathered with the help of EU-RHAB. Time to event was calculated as the time from the first diagnosis of AT/RT until the time of death. Censoring was noticed at the last time of contact if the patient was still alive. Survival analysis was performed based on bulk RNAseq data of a cohort of AT/RT-MYC patients (Table S6). At first, the cohort was separated into one group expressing a high number of in total 137 upregulated genes identified in primary tumor clusters 2, 4, and 5 (Table S3), with a log_2_ gene expression > 2, and one group expressing a low number of these genes. The cutoff between these two groups was calculated using the *maxstat.test()* function of the *maxstat* package (version 0.7-25, RRID:SCR_025679) [26] in R, which performs a Log-Rank test and calculates the optimal separation between two groups. To investigate the influence of covariates, a multivariate analysis was performed using the *coxph()* function of the survival package (version 3.6.4, RRID:SCR_021137) [55] with age and sex as covariates. The resulting hazard ratios were visualized using the *forestmodel* package (version 0.6.2, RRID:SCR_025250) [31]. For further analyses, only patients with a minimum follow-up of 17 months were selected and separated into long and short survivors at the median. For the TME analysis, bulk RNAseq data of AT/RT patients of all DNA methylation subtypes were stratified into two groups based on the abundance of *CD1A*^+^ *CD207*^+^ cells in the respective recurrent tumor samples as described above. The abundance of *CD1A*^+^ *CD207*^+^ DCs was defined as follows: For IHC, samples with ≥ 0.1% CD1A-/CD207-positive area were defined as abundant. For bulk RNAseq, samples with a *CIBERSORT* score ≥ 0.001 were defined as abundant. For snRNAseq, samples with cells present in neighborhood group 9 were defined as abundant. Kaplan–Meier curves [30] were generated using the *ggsurvfit* package (version 1.1.0, RRID:SCR_025045) [52]. Comparisons of survival curves were performed via two-sided Log-Rank tests, with *p* < 0.05 being significant using the package *survival* (version 3.6.4, RRID:SCR_021137) [55]. To identify DEG and pathways of long vs. short survivors, the *DESeq2* package (version 1.46.0, RRID:SCR_015687) [36] was used, and all genes with a *p *value < 0.05 after BH adjustment and a log_2_ fold change > 1 were chosen. Again, the *Reactome Pathway* database was used for gene set enrichment analysis using the *gsePathway()* function included in the *ReactomePA* package (version 1.50.0, RRID:SCR_019316) [65]. Results were visualized in a *dotplot* with the *enrichplot* package (version 1.26.6, RRID:SCR_018931) [64].

### Statistics

For differential gene expression analysis, two-sided *t *tests were used (Figs. 2j, 3c, d, 4e, f, 5c, d). To identify differences between cancer cell states, two-sided paired *t *tests were used (Fig. 4a). EMA IHC quantification was performed using one-sided (Fig. 4c) and two-sided paired *t *tests (Fig. 4d). Significant differences of survival curves were determined using two-sided Log-Rank tests (Fig. 5a, b). Correction for multiple testing was done using BH adjustment, and *p* < 0.05 was considered to indicate statistical significance.

## Results

### Single-nucleus RNA sequencing (snRNAseq) analysis of eight paired primary and recurrent AT/RT demonstrated distinct gene expression profiles

**Fig. 1:**
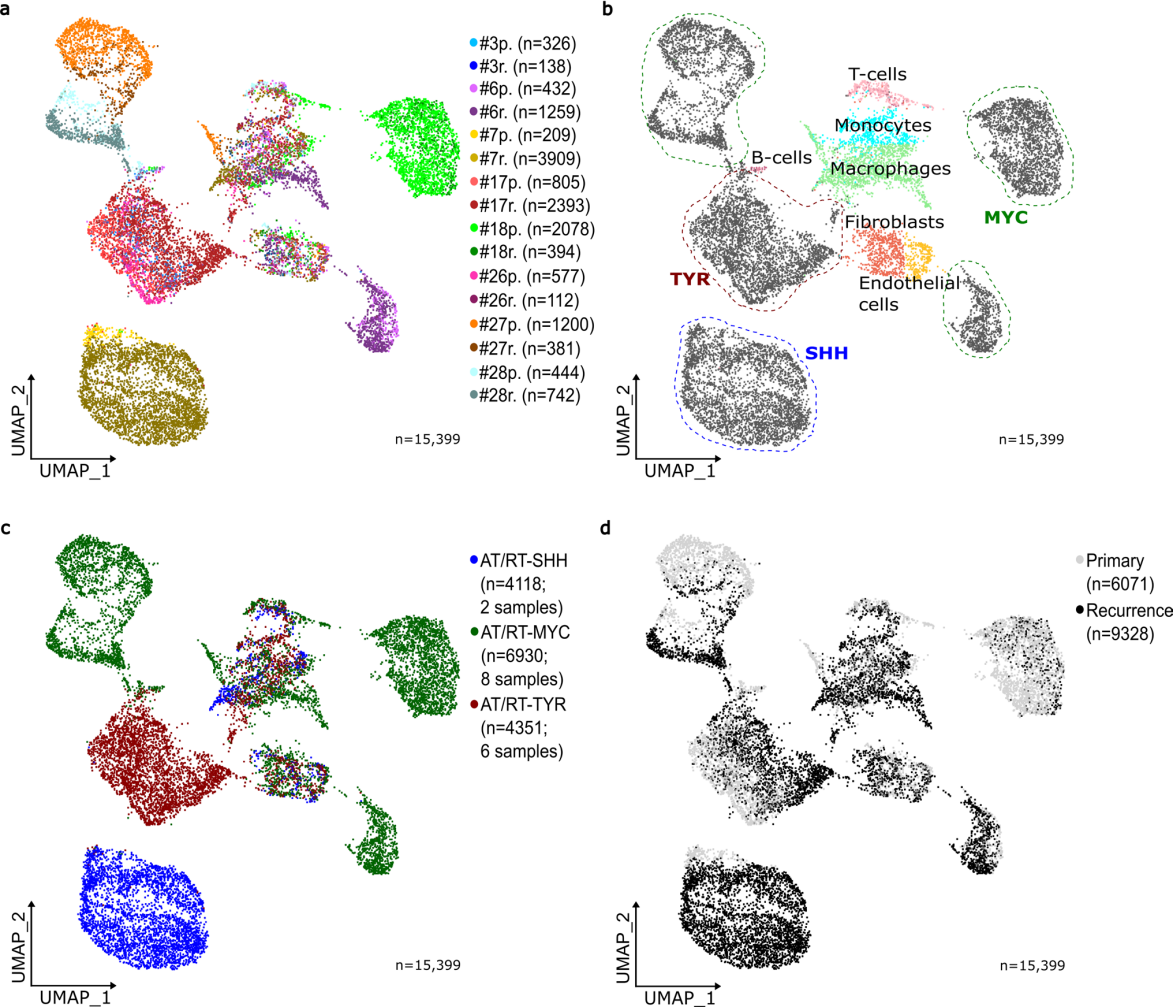
Overview of all samples and UMAPs of the snRNAseq data of all primary and recurrent AT/RT, including tumor cells and TME. UMAP using 10 neighboring points of *SCT*-normalized counts of all cells (*n* = 15,399), annotated by samples (**a**). UMAP of all cells (*n* = 15,399), with annotated TME and DNA methylation subtypes assigned (**b**). UMAP of all cells (*n* = 15,399), annotated by DNA methylation subtypes (**c**). UMAP of all cells (*n* = 15,399), annotated by status (primary or recurrence) (**d**)

snRNAseq was performed for 16 samples from eight patients (Fig. S1a–S1d, Table S1). The *SCT*-normalized dataset comprising all samples was visualized using a UMAP for dimension reduction (*n* = 15,399 cells), resulting in distinct clusters for the tumor cells and the tumor microenvironment (TME), which could be distinguished by CNVs of chromosome 22 (Fig. S1e), chromosome 10 (Fig. S1f), and chromosome 1 (Fig. S1g). The TME was characterized by clusters of cells from all samples, which were assigned to their respective cell types (Figs. 1a, b, S2a–S2b, Table S2). To decipher compositional changes in TME populations, we performed differential abundance testing, which identified a significantly enriched abundance of *CD1A*^+^ *CD207*^+^ dendritic cells (DCs) in recurrences (Fig. S2c–S2h). This finding was validated by CD1A and CD207 immunohistochemistry (IHC; Fig. S3a, S3b) and a deconvolution of previously published bulk RNAseq data (Fig. S3c, S3d) [28]. In addition, patients suffering from tumors presenting *CD1A*^+^ *CD207*^+^ DCs had a significantly shorter overall survival (OS) compared to patients without detection of such cells (*p* = 0.009, two-sided Log-Rank test; Fig. S3e). Cell–cell communication inference via *nichenetr* identified an enriched *TGF-β1*- and *TGF-β2*-induced signaling in recurrent AT/RT (Fig. S3f, S3g) as a potential cause for the observed *CD1A*^+^ *CD207*^+^ DCs. The tumor cells of AT/RT-MYC (*n* = 4 patients) demonstrated patient-specific differences, whereas AT/RT-TYR tumor cells of all patients (*n* = 3 patients) were mixed in one cluster, indicating more similar gene expression profiles of single AT/RT-TYR tumor samples compared to the other AT/RT types (Figs. 1c, S1h). Nevertheless, distinct gene expression profiles for each AT/RT type were observed (Fig. S1e). Paired primary and recurrent tumor samples showed distinct gene expression profiles (Figs. 1d, S1i).

### AT/RT-MYC primary tumor cells with high similarity to recurrences are characterized by altered ECM organization, developmental processes, and immune system pathways

**Fig. 2:**
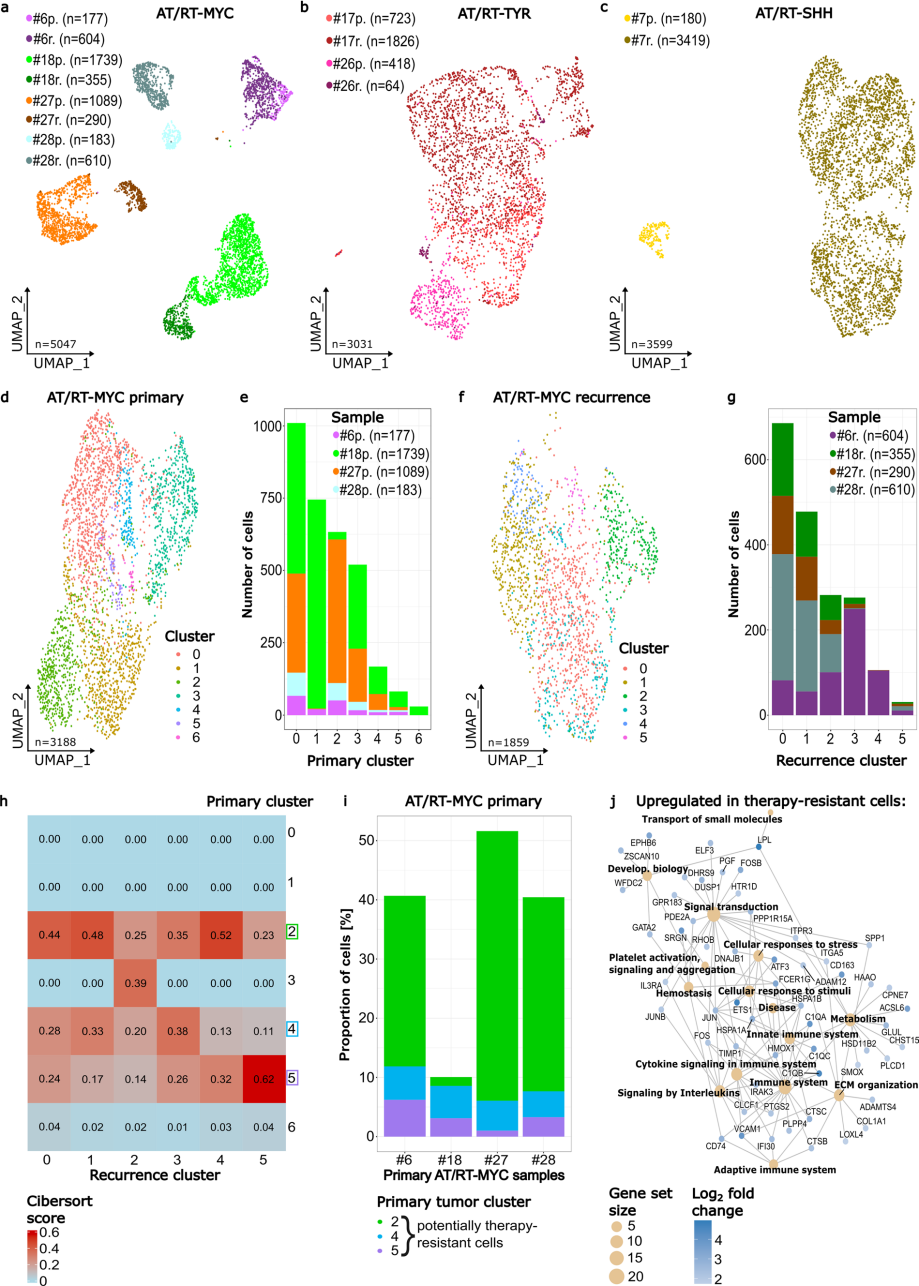
Analysis of tumor cells only and identification of AT/RT-MYC primary tumor cells with high similarity to recurrences. UMAP using 10 neighboring points of *SCT*-normalized counts of AT/RT-MYC tumor cells only (*n* = 5047), annotated by samples (**a**). UMAP of AT/RT-TYR tumor cells only (*n* = 3031), annotated by samples (**b**). UMAP of AT/RT-SHH tumor cells only (*n* = 3599), annotated by samples (**c**). Integrated UMAP using 10 neighboring points of all AT/RT-MYC primary tumor cells (*n* = 3188), annotated by *Seurat* clusters (**d**). Bar plot showing the distribution of all MYC primary tumor cells over the primary tumor clusters, annotated by sample (**e**). Integrated UMAP of all AT/RT-MYC recurrence tumor cells (*n* = 1859), annotated by *Seurat* clusters (**f**). Bar plot showing the distribution of all MYC recurrence tumor cells over the recurrence tumor clusters, annotated by sample (**g**). Heatmap of *CIBERSORT* results for investigating similarities between AT/RT-MYC primary tumor clusters and recurrence tumor clusters, showing highest *CIBERSORT* scores for primary tumor clusters 2, 4, and 5 (**h**). Bar plot showing the distribution of the potentially therapy-resistant AT/RT-MYC primary tumor clusters 2, 4, and 5 over the primary tumor samples (**i**). Upregulated genes and *Reactome* pathways in potentially therapy-resistant AT/RT-MYC primary tumor cells (primary tumor clusters 2, 4, and 5) compared to all other primary tumor cells (**j**)

The AT/RT-MYC primary and recurrent tumor cells of cases 6, 18, 27, and 28 demonstrated distinct gene expression profiles (Fig. 2a, Table S3, Fig. S1e, S1f). For AT/RT-TYR, case 3 was excluded due to a small number of tumor cells. The primary and recurrent tumor cells of cases 17 and 26 showed more similar but still distinct gene expression profiles compared to AT/RT-SHH and AT/RT-MYC (Figs. 2b, S1e, S1f). Primary and recurrent AT/RT-SHH tumor cells showed a clear separation in the UMAP (Fig. 2c). Due to the relatively low number of samples and cells belonging to AT/RT-TYR and AT/RT-SHH, further analyses focused on AT/RT-MYC tumor cells. A stronger integration of the snRNAseq data of the AT/RT-MYC tumor cells was performed in order to identify molecular mechanisms of tumor progression and recurrence occurring in all patients. This was done for all primary and recurrent tumor cells separately. After that, seven *Seurat* clusters were identified for the primary tumor cells (Fig. 2d, e) and six *Seurat* clusters for the recurrences (Fig. 2f, g). Using *CIBERSORT*, primary tumor clusters 2, 4, and 5 revealed the highest similarity to the recurrences (Figs. 2h, S4a). Quantifying the cells belonging to these clusters for each sample confirmed the presence of such cells in all samples (Fig. 2i). The top 15 upregulated *Reactome* pathways and associated genes in the cells were identified, with altered ECM organization as a top feature. Furthermore, immune system-associated pathways were upregulated, like increased cytokine signaling as well as alterations in the adaptive and innate immune system (Fig. 2j, Table S3). Finally, a number of upregulated genes were involved in metabolic processes, signal transduction, hemostasis, and developmental processes. Similar genes and pathways were upregulated in AT/RT-MYC recurrences compared to primary tumors (Fig. S4b, S4c).

### Potentially therapy resistance-associated genes were less expressed in AT/RT-MYC that did not relapse

**Fig. 3:**
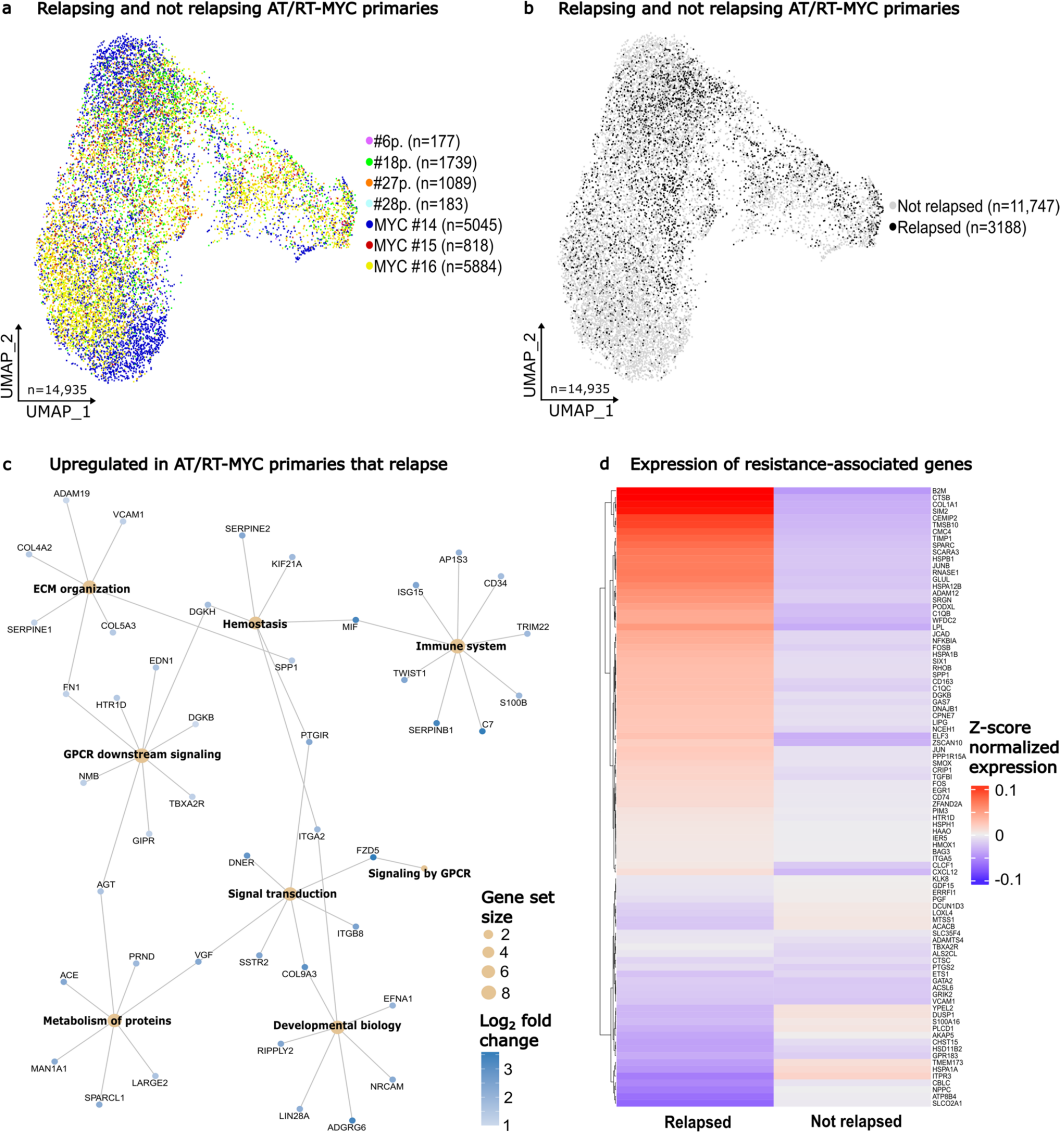
Integration of AT/RT-MYC primary tumors that did not relapse and investigation of the expression of therapy resistance-associated genes. Integrated UMAP using 10 neighboring points of normalized counts of AT/RT-MYC primary tumors that relapsed and that did not relapse (*n* = 14,935), annotated by samples (**a**). Integrated UMAP of AT/RT-MYC primary tumors that relapsed and that did not relapse (*n* = 14,935), annotated by status (**b**). Upregulated genes and *Reactome* pathways in AT/RT-MYC primary tumors that relapsed compared to the tumors that did not relapse until now (**c**). Heatmap showing *Z*-score normalized expression of genes identified in potentially therapy-resistant AT/RT-MYC cells (primary tumor cluster 2, 4, and 5). The mean expression for all relapsing and not relapsing AT/RT-MYC was calculated and visualized (**d**)

To validate whether gene signatures identified in potentially therapy-resistant AT/RT-MYC cells (primary tumor cluster 2, 4, and 5; Fig. 2j) may be associated with the development of recurrences, we analyzed the expression of respective genes in AT/RT-MYC tumors that did not relapse (Table S6). We performed *CCA* integration to add these samples to our cohort of AT/RT-MYC primary tumors that relapsed (*n* = 14,935 cells; Fig. 3a, b). Upregulated genes and pathways in AT/RT-MYC primary tumors that relapsed were visualized in Fig. 3c, which also include altered ECM organization, immune system pathways, and developmental processes, among others. Furthermore, we investigated the expression of genes identified in potentially therapy-resistant cells. The majority of respective genes were upregulated in AT/RT-MYC tumors that relapsed compared to those that did not relapse (Fig. 3d).

### AT/RT-MYC recurrences contained an increased proportion of cells undergoing pEMT as compared to primary tumors

**Fig. 4:**
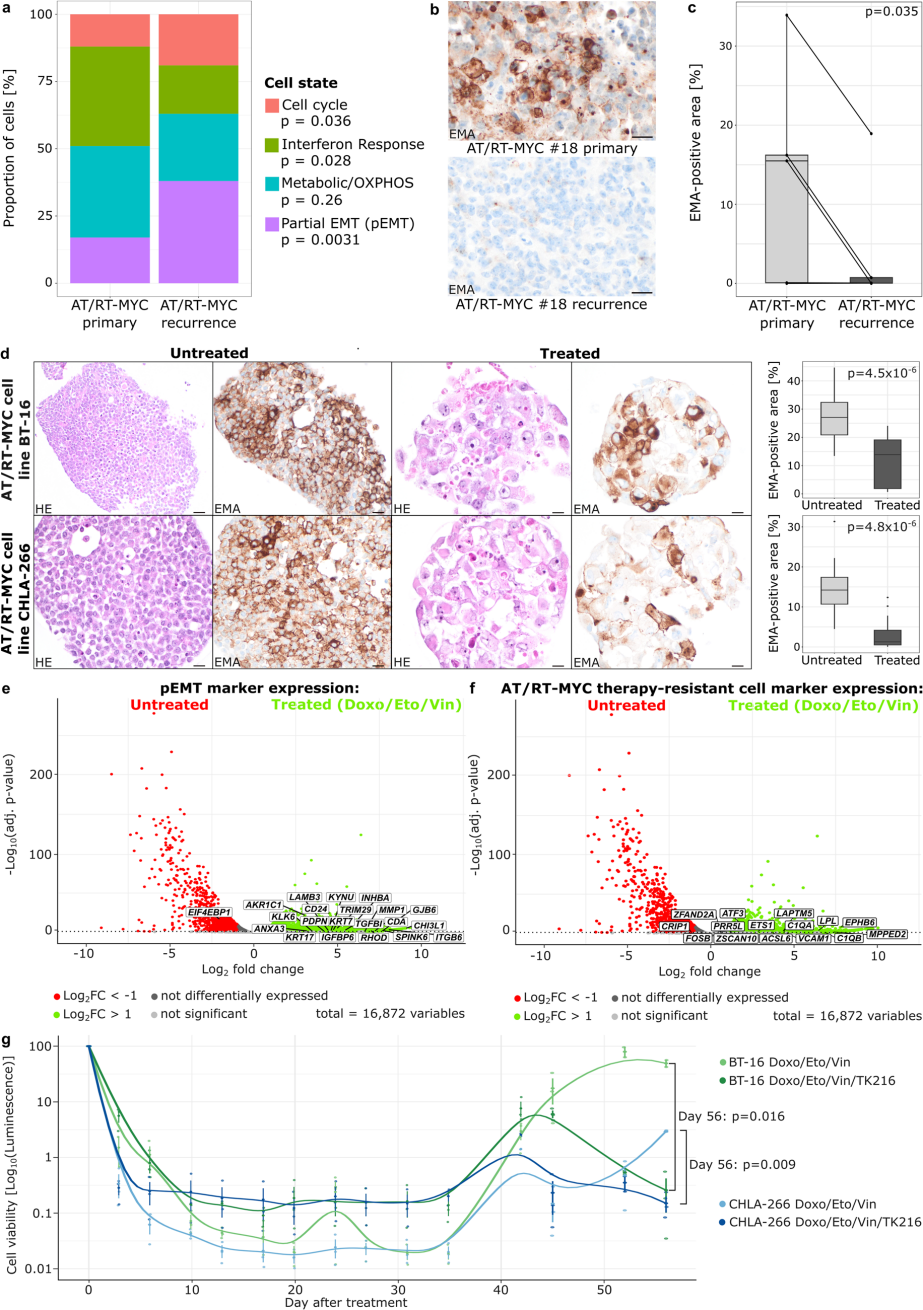
Cancer cell states of AT/RT-MYC tumor cells and in vitro validation using patient-derived AT/RT-MYC cell lines. Distribution of cancer cell states of integrated AT/RT-MYC recurrence tumor cells compared to AT/RT-MYC primary tumor cells, resulting in an increased proportion of cells undergoing pEMT (*p* = 0.0031, two-sided paired *t*-test). *OXPHOS* oxidative phosphorylation, *pEMT* partial epithelial to mesenchymal transition (**a**). IHC staining of the EMA protein, exemplary for AT/RT-MYC case 18 (primary tumor and paired recurrence). Scale bars indicate 20 µm (**b**). Quantification of EMA IHC for five AT/RT-MYC primary-recurrence pairs resulted in decreased EMA-positive area in recurrences for three pairs, two pairs showed similar EMA expression (*p* = 0.035, one-sided paired *t*-test) (**c**). In vitro validation of the distribution of epithelial cells using two AT/RT-MYC cell lines: BT-16 and CHLA-266. H&E and IHC EMA staining was performed before and after treatment with a combination of doxorubicin (doxo), etoposide (eto), and vincristine (vin). Quantification of the EMA staining resulted in decreased EMA-positive area after the treatment BT-16 (*p* = 4.5 × 10^−6^, two-sided paired *t*-test) and CHLA-266 (*p* = 4.8 × 10^−6^, two-sided paired *t*-test) Scale bars indicate 20 µm (**d**). Volcano Plot of bulk RNAseq data showing DEG of BT-16 and CHLA-266 untreated (red) vs. BT-16 and CHLA-266 treated with doxorubicin, etoposide, and vincristine (green) with pEMT marker genes annotated [5]. Most of the pEMT marker genes were upregulated after treatment (**e**). Volcano Plot of bulk RNAseq data showing DEG of BT-16 and CHLA-266 untreated (red) vs. BT-16 and CHLA-266 treated with doxorubicin, etoposide, and vincristine (green) with marker genes of potentially therapy-resistant AT/RT-MYC primary tumor cells (primary tumor cluster 2, 4, and 5) annotated. Most of the marker genes of potentially therapy-resistant cells were upregulated after treatment (**f**). Cell viability assay resulted in decreased therapy resistance for BT-16 (*p* = 0.016, two-sided paired *t*-test) and CHLA-266 (*p* = 0.009, two-sided paired *t*-test) treated with TK216 in addition to doxo, eto, and vin compared to standard therapy (doxo, eto, vin; **g**)

To further characterize molecular differences between primary and recurrent AT/RT-MYC tumor cells, each cell was assigned to a cancer cell state using non-negative matrix factorization (NMF) analysis, which resulted in four different cancer cell states, which were annotated as “cell cycle”, “interferon response”, “metabolic/oxidative phosphorylation (OXPHOS)”, and “partial epithelial to mesenchymal transition (pEMT)” (Table S4). Quantification resulted in an increased number of AT/RT-MYC recurrence cells undergoing pEMT (*p* = 0.0031, two-sided paired *t*-test, Figs. 4a, S5a–S5d). Increased pEMT in recurrences was also observed using a pan-cancer cell state atlas [5] as a control (Fig. S5f, S5g). This finding was further investigated by using antibodies against EMA for IHC of paired AT/RT-MYC primary tumors and recurrences (Figs. 4b, S5e). Quantification of the EMA-positive area for three pairs resulted in a decreased EMA expression in AT/RT-MYC recurrences in three out of five pairs with two out of five pairs showing similar EMA expression (*p* = 0.035, one-sided paired *t*-test, Fig. 4c). We then treated two AT/RT-MYC cell lines using doxo, eto, and vin on three consecutive days with subsequent regrowth for investigating changes in the proportion of pEMT. H&E and EMA stainings were done for each cell line before and after treatment, resulting in a decreased EMA expression after treatment for BT-16 (*p* = 4.5 × 10^−6^, two-sided paired *t*-test) and CHLA-266 (*p* = 4.8 × 10^−6^, two-sided paired *t*-test, Fig. 4d). Investigation of the H&E stainings revealed a lower cell density and larger distances between the cells after treatment. Bulk RNAseq confirmed our observations by resulting in DEG between treated and untreated cells (Fig. S5h, Table S5), including an upregulated gene expression of pEMT marker genes (Fig. 4e). Six mesenchymal marker genes (*SNAI2*, *MMP9*, *ZEB2*, *SOX10*, *FN1*, and *ACTA2*) were also upregulated after treatment (Fig. S5i). In addition, the top 15 upregulated genes of AT/RT-MYC primary tumor clusters 2, 4, and 5, containing the potentially therapy-resistant cells, were almost all increased in both cell lines after treatment (Fig. 4f). As *ETS1*, a gene associated with therapy-resistance and pEMT, was upregulated in potentially therapy-resistant primary tumor cells (Fig. 2j) as well as in therapy-resistant AT/RT-MYC cell lines (Fig. 4i), we investigated whether the ETS1 inhibitor TK216 could potentiate the therapy response. Cell viability of BT-16 cells treated with doxo, eto, vin, and TK216 was significantly lower compared to BT-16 cells treated with doxo, eto, and vin alone at day 56 after treatment (*p* = 0.016, two-sided paired *t*-test). A similar effect was observed in CHLA-266 cells treated with a combination of doxo, eto, vin, and TK216 compared to cells without TK216 (*p* = 0.009, two-sided paired *t*-test; Fig. 4g).

### AT/RT-MYC patients with tumors overexpressing genes identified in potentially therapy-resistant cells showed worse survival

**Fig. 5:**
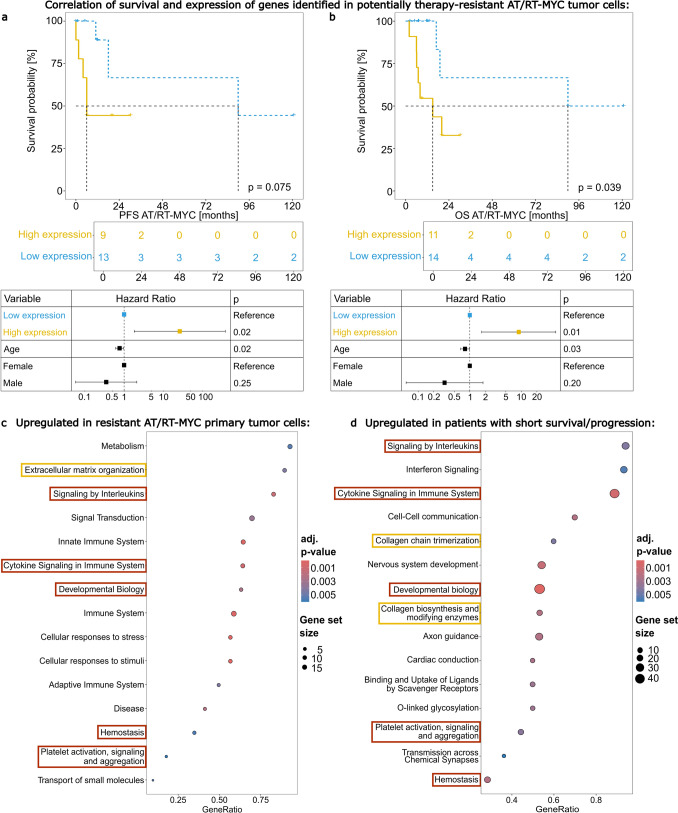
Survival analysis of AT/RT-MYC patients in correlation with the expression of genes identified in potentially therapy-resistant primary tumor cells. PFS of a cohort of AT/RT-MYC patients separated into two groups using the best cutoff. There was no difference in the PFS of patients with an overexpression of a high number of genes identified in potentially therapy-resistant AT/RT-MYC primary tumor cells (primary tumor clusters 2, 4, and 5; *n* = 9) compared to the patients with a low number of these genes (*n* = 13, *p* = 0.075, two-sided Log-Rank test). Multivariate analysis resulted in significant inferior PFS of patients with overexpression of a high number of such genes (hazard ratio = 26.2, *p* = 0.02, two-sided Log-Rank test). Younger age was correlated with inferior PFS (hazard ratio = 0.77, *p* = 0.02, two-sided Log-Rank test) (**a**). OS of a cohort of AT/RT-MYC patients separated into two groups using the best cutoff. Patients with an overexpression of a high number of genes identified in potentially therapy-resistant AT/RT-MYC primary tumor cells (primary tumor clusters 2, 4, and 5; *n* = 11) had a significantly worse OS compared to the patients with a low number of these genes (*n* = 14, *p* = 0.039, two-sided Log-Rank test). Multivariate analysis resulted in significant inferior OS of patients with overexpression of a high number of such genes (hazard ratio = 8.91, *p* = 0.01, two-sided Log-Rank test). Younger age was correlated with inferior OS (HR 0.81, *p* = 0.03, two-sided Log-Rank test) (**b**). Dot plot of the top 15 *Reactome* pathways upregulated in potentially therapy-resistant AT/RT-MYC primary tumor cells (primary tumor clusters 2, 4, and 5). The size of the dots indicated the gene set size, and the dots were colored according to their adjusted *p *value. The pathways marked in red were overlapping pathways with d. The pathways marked in orange had similar functions to pathways in d (**c**). Dot plot of the top 15 *Reactome* pathways upregulated in AT/RT-MYC patients with short survival and/or disease progression (according to **c** and **d**). The size of the dots indicated the gene set size, and the dots were colored according to their adjusted *p*-value. The pathways marked in red were overlapping pathways with **c**. The pathways marked in orange had similar functions to pathways in **c** (**d**)

Next, we investigated the clinical impact of our findings. To this end, we collected a cohort of bulk RNAseq data of 25 AT/RT-MYC primary tumors (Table S6). We separated the cohort into one group expressing a high number of genes identified in a subset of primary tumor cells (primary tumor clusters 2, 4, and 5) as well as in recurrences and another group expressing a low number of these genes. Age and sex distributions were equal in these two groups, and no differences between the resection and metastases status as well as the applied therapy protocols were observed (Table S6). Patients with tumors highly expressing the recurrence-associated gene signature showed inferior OS (*p* = 0.039, two-sided Log-Rank test, Fig. 5a, b). In addition, multivariate analysis resulted in significant inferior OS for AT/RT-MYC patients whose tumors exhibited high expression of therapy resistance-associated genes compared to those with low expression (hazard ratio = 8.91, *p* = 0.01). This was similar for the PFS (hazard ratio = 26.2, *p* = 0.02). Moreover, younger age was also correlated with inferior survival (OS: Hazard ratio 0.81, *p* = 0.02; PFS: Hazard ratio 0.77, *p* = 0.03; Fig. 5a, b). With each 1-year increase in age, the risk of death decreases by 19% and the risk of progression decreases by 23%. Furthermore, we compared the gene expression profiles of long vs. short survivors by using the same cohort of bulk RNAseq data. Upregulated pathways in relapsing AT/RT-MYC tumor cells (primary tumor clusters 2, 4, and 5) and recurrences (Fig. 5c) were compared to upregulated pathways in short survivors (Fig. 5d). The top 15 pathways were visualized, resulting in five out of 15 overlapping pathways (highlighted in red). These were cytokine signaling, including signaling by interleukins, developmental biology, and hemostasis, including platelet activation, signaling, and aggregation. In addition, the pathways collagen chain trimerization and collagen biosynthesis and modifying enzymes are characterized by similar gene sets and functions as the *Reactome* pathway ECM organization. In summary, increased cytokine signaling, developmental processes, hemostasis, and ECM organization were not only associated with primary AT/RT-MYC tumor cells being highly similar to recurrences and potentially therapy-resistant but also correlated with poor survival in AT/RT-MYC patients. In addition, the top five upregulated genes in therapy-resistant cells, *ETS1*, *LAPTM5*, *LPL*, *CRIP1*, and *C1QB,* were investigated. For *LPL*, *CRIP1*, and *C1QB*, no significant survival differences were observed, but high *ETS1* expression was correlated to inferior progression-free survival (PFS) and OS of AT/RT-MYC patients (*p* = 0.00054; *p* = 0.00058; Fig. S6a, S6b). High expression of *LAPTM5* was correlated to inferior PFS of AT/RT-MYC patients (*p* = 0.0049; Fig. S6c, S6d).

## Discussion

Previously, we have identified molecular differences of paired primary and recurrent AT/RT on DNA methylation, bulk gene expression, and histologic level [29]. However, for the identification of exact molecular mechanisms and novel therapeutic targets, more detailed analyses were required. Our snRNAseq analysis confirmed the existence of molecular differences between primary and recurrent AT/RT, not only in the tumor cells, but also in the TME. Additionally, potentially therapy-resistant cells and promising therapeutic targets were identified, shedding light on mechanisms of tumor progression and recurrence.

snRNAseq data were separated into tumor cells and TME based on TME marker genes and CNVs. To decipher compositional changes in TME cell populations, we performed differential abundance testing. The cells enriched in AT/RT recurrences were identified as DCs differentiated similarly to Langerhans cells (LHCs) [35]—a previously undescribed feature in TME landscapes of CNS tumors. The highest proportion of respective cells was found in the recurrence of case 17, which matched the CD1A and CD207 IHC quantification results. Cell–cell communication inference revealed that *TGF-β1*- and *TGF-β2*-induced signaling might activate these *CD1A*^+^ *CD207*^+^ DCs in AT/RT recurrences, a finding consistent with previous studies on LHCs, which was also demonstrated by in vitro purification of such cells from different progenitors via *TGF-β* [62]. In addition, patients with *CD1A*^+^ *CD207*^+^ DC enrichment in AT/RT recurrences had a shorter OS, which was independent of the subtype distribution between the two groups. However, for consideration as a therapeutic target, more samples for snRNAseq analyses must be collected to validate our findings.

To further identify resistance mechanisms, AT/RT-MYC primary tumor cells with high similarity to recurrences were identified over all AT/RT-MYC samples. We hypothesize that these cells persist following therapy and develop to recurrences. In general, it is assumed that tumor recurrences arise from dormant tumor cells, characterized by drug resistance and immune escape [11]. Due to the high similarity to AT/RT-MYC recurrences, we assumed that primary tumor clusters 2, 4, and 5 contain such therapy-resistant cells. They were characterized by an altered ECM organization, among other features. The ECM is generally known to support tumor progression in different tumor entities, by acting as a biomechanical barrier and by protecting the tumor from anticancer agents [61]. In addition, different therapies induce ECM remodeling, leading to the occurrence of therapy-resistant cells. These findings make the ECM a promising target for novel therapies [61]. Furthermore, we observed increased cytokine signaling, which is also associated with tumor progression in different entities [63]. On the one hand, cytokines can induce EMT and the development to dormant tumor cells [63]. Both EMT and dormant tumor cells play an important role in therapy resistance and tumor progression. On the other hand, cytokine signaling is involved in the ECM remodeling through cancer-associated fibroblast activation [63]. Furthermore, imbalanced hemostasis was associated with tumor progression, especially due to increased platelet aggregation [15], whereas altered developmental processes correlate with EMT by regulating the fate of embryonic and malignant cells. Additionally, it is associated with tumor progression and recurrence in diverse entities [57]. Altered ECM organization increased cytokine signaling, and altered developmental processes were also characteristics of AT/RT-MYC recurrences compared to primary tumors, which confirms the similarity of the identified potentially therapy-resistant cells to AT/RT-MYC recurrences.

Another confirmation of our hypothesis was that potentially therapy resistance-associated genes were less expressed in AT/RT-MYC tumors that did not relapse. Three AT/RT-MYC patients, who did not show any indications of tumor progression, demonstrated a significantly lower expression of respective genes. This confirmed that overexpression of the identified genes drives AT/RT-MYC tumor progression and development of recurrences.

To better understand transcriptional heterogeneity as a driver of tumor progression, recurrence, and failure of existing therapies [3], we assigned each AT/RT-MYC tumor cell to a cancer cell state using NMF analysis, which revealed increased pEMT in AT/RT-MYC recurrences. The cancer cell state allocation was confirmed by using a pan-cancer cell state atlas [5], which also resulted in increased pEMT in recurrences. PEMT refers to the transition phase from epithelial to mesenchymal cells, whereas a complete EMT (cEMT) was defined by a complete transition to mesenchymal cells, and therefore, the expression of mesenchymal marker genes [5]. PEMT is a hybrid state with tumor cells expressing both specific epithelial and mesenchymal marker genes [48]. As described above, pEMT is associated with tumor progression and recurrence in many ways. Therefore, we investigated epithelial genes using IHC. Treated AT/RT-MYC cell lines were not only showing a decreased expression of the epithelial marker EMA, they were also characterized by an altered morphology with lose cell–cell contacts [47]. Bulk RNAseq confirmed our observations. Not only pEMT marker genes were upregulated in the cells after treatment, in addition, the top upregulated genes in therapy-resistant tumor cells (identified by snRNAseq analysis) were overexpressed. Previously, expression of both epithelial markers (EMA) and mesenchymal markers [vimentin and smooth muscle actin (SMA)] was detected in nearly all investigated AT/RT cases [46, 47] and co-expression of both marker types was observed in subsets of cells [9]. Our findings suggest that these are not only characteristic histopathological features but also indicate that pEMT plays a general role in AT/RT, given the frequent co-existence of epithelial and mesenchymal characteristics. This co-existence, which has been associated with pEMT [48], may contribute to the aggressiveness and therapy resistance of AT/RT. As mesenchymal areas were enriched in AT/RT-MYC [66], we hypothesize that pEMT plays a particularly important role in this subtype, which has previously been associated with an unfavorable prognosis (non-TYR subtype) [17]. To investigate whether inhibition of pEMT improves therapeutic response by reducing therapy resistance, we analyzed the effect of the ETS1 inhibitor TK216, as *ETS1* was overexpressed in potentially therapy-resistant AT/RT-MYC primary tumor cells as well as in therapy-resistant AT/RT-MYC cell lines. *ETS1* has previously been characterized as a negative prognostic marker in carcinomas, including squamous cell carcinomas, and may play a crucial role in invasiveness, EMT, ECM, and therapy resistance [49, 50], consistent with our observations in AT/RT-MYC. Inhibition of *ETS1* has been shown to enhance sensitivity to anti-tumor drugs in squamous cell carcinoma cells [49]. Similar effects have been reported for the small molecule inhibitors YK-4-279 and its clinically improved derivative TK216 in Ewing sarcoma [42], pediatric leukemia [51], and lymphoma [53]. TK216 is currently being tested in a phase 2 clinical trial for relapsed or refractory Ewing sarcoma (NCT05046314). In our study, the combination of standard chemotherapy (doxo, eto, and vin) with TK216 resulted in significantly reduced regrowth. In conclusion, inhibition of pEMT via ETS1 targeting decreased therapy resistance in AT/RT-MYC cell lines.

Finally, we observed a correlation between overexpression of the genes identified in potentially therapy-resistant cells and the OS of AT/RT-MYC patients. These survival differences were independent of age, although age has been shown to have an impact on survival. However, the median age of the group with inferior survival (6.3 years) was higher than that of the group with better survival (2.9 years). This confirmed our hypothesis that the occurrence of therapy-resistant cells is associated with tumor progression and recurrence, and therefore, with an inferior prognosis. We further investigated the top five upregulated genes in therapy-resistant cells. High *ETS1* and *LAPTM5* expression was correlated to inferior survival of AT/RT-MYC patients. As described above, *ETS1* is involved in therapy resistance and EMT. In addition, *ETS1* expression is regulated by *TGF-β* [49] which we previously identified in the context of *CD1A*^+^ *CD207*^+^ DC activation, and therefore, *TGF-β* seems to play a role in both tumor cells and TME alterations regarding tumor progression and recurrence. The lysosomal transmembrane protein 5 (*LAPTM5*) was previously found to be increased in the context of tumor progression in different entities [54] and could be another target for AT/RT-MYC therapy. *LAPTM5* was recently found to be associated with shorter OS in glioblastomas, there facilitating chemotherapy resistance in glioblastomas [6].

Overall, we identified promising molecular characteristics of therapy resistance in AT/RT, including increased ECM remodeling and pEMT, which should be considered as targets for novel therapeutic approaches.

## Supplementary Information

Below is the link to the electronic supplementary material.

Supplementary file1 (PDF 83018 KB)

Supplementary file2 (XLSX 420 KB)

## Data Availability

All generated data of this study are deposited at NCBI Gene Expression Omnibus (GEO; https://www.ncbi.nlm.nih.gov/geo). The snRNAseq data of paired primary and recurrent AT/RT are accessible through GEO Series accession number GSE298139. Bulk RNAseq data of AT/RT-MYC primary tumors were obtained from previous studies [28, 30] or were generated for this study. These data are accessible through GEO Series accession number GSE298140. Bulk RNAseq data of the untreated and treated AT/RT-MYC cell lines are accessible via GSE298141.

## References

[1] Aibar S, González-Blas CB, Moerman T, Huynh-Thu VA, Imrichova H, Hulselmans G et al (2017) SCENIC: single-cell regulatory network inference and clustering. Nat Methods 14:1083–1086. 10.1038/nmeth.4463 28991892 10.1038/nmeth.4463 PMC5937676

[2] Aleksander SA, Balhoff J, Carbon S, Cherry JM, Drabkin HJ, Ebert D et al (2023) The Gene Ontology knowledgebase in 2023. Genetics. 10.1093/genetics/iyad031 36866529 10.1093/genetics/iyad031 PMC10158837

[3] Alizadeh AA, Aranda V, Bardelli A, Blanpain C, Bock C, Borowski C et al (2015) Toward understanding and exploiting tumor heterogeneity. Nat Med 21:846–853. 10.1038/nm.3915 26248267 10.1038/nm.3915 PMC4785013

[4] Ashburner M, Ball CA, Blake JA, Botstein D, Butler H, Cherry JM et al (2000) Gene Ontology: tool for the unification of biology. Nat Genet 25:25–29. 10.1038/75556 10802651 10.1038/75556 PMC3037419

[5] Barkley D, Moncada R, Pour M, Liberman DA, Dryg I, Werba G et al (2022) Cancer cell states recur across tumor types and form specific interactions with the tumor microenvironment. Nat Genet 54:1192–1201. 10.1038/s41588-022-01141-9 35931863 10.1038/s41588-022-01141-9 PMC9886402

[6] Berberich A, Bartels F, Tang Z, Knoll M, Pusch S, Hucke N et al (2020) LAPTM5-CD40 crosstalk in glioblastoma invasion and temozolomide resistance. Front Oncol 10:747. 10.3389/fonc.2020.00747 32582531 10.3389/fonc.2020.00747 PMC7289993

[7] Biegel JA, Zhou JY, Rorke LB, Stenstrom C, Wainwright LM, Fogelgren B (1999) Germ-line and acquired mutations of INI1 in atypical teratoid and rhabdoid tumors. Cancer Res 59:74–79 9892189

[8] Blighe K, Rana S, Lewis M (2025) EnhancedVolcano: publication-ready volcano plots with enhanced colouring and labeling. The Comprehensive R Archive Network

[9] Bouffard J-P, Sandberg GD, Golden JA, Rorke LB (2004) Double immunolabeling of central nervous system atypical teratoid/rhabdoid tumors. Mod Pathol 17:679–683. 10.1038/modpathol.3800099 15105808 10.1038/modpathol.3800099

[10] Browaeys R, Saelens W, Saeys Y (2020) NicheNet: modeling intercellular communication by linking ligands to target genes. Nat Methods 17:159–162. 10.1038/s41592-019-0667-5 31819264 10.1038/s41592-019-0667-5

[11] Damen MPF, van Rheenen J, Scheele CLGJ (2021) Targeting dormant tumor cells to prevent cancer recurrence. FEBS J 288:6286–6303. 10.1111/febs.15626 33190412 10.1111/febs.15626

[12] Dann E, Henderson NC, Teichmann SA, Morgan MD, Marioni JC (2022) Differential abundance testing on single-cell data using k-nearest neighbor graphs. Nat Biotechnol 40:245–253. 10.1038/s41587-021-01033-z 34594043 10.1038/s41587-021-01033-z PMC7617075

[13] Dobin A, Davis CA, Schlesinger F, Drenkow J, Zaleski C, Jha S et al (2013) STAR: ultrafast universal RNA-seq aligner. Bioinformatics 29:15–21. 10.1093/bioinformatics/bts635 23104886 10.1093/bioinformatics/bts635 PMC3530905

[14] Durinck S, Spellman PT, Birney E, Huber W (2009) Mapping identifiers for the integration of genomic datasets with the R/Bioconductor package biomaRt. Nat Protoc 4:1184–1191. 10.1038/nprot.2009.97 19617889 10.1038/nprot.2009.97 PMC3159387

[15] Falanga A, Marchetti M (2018) Hemostatic biomarkers in cancer progression. Thromb Res 164:S54–S61. 10.1016/j.thromres.2018.01.017 29703485 10.1016/j.thromres.2018.01.017

[16] Franzén O, Gan L-M, Björkegren JLM (2019) PanglaoDB: a web server for exploration of mouse and human single-cell RNA sequencing data. Database. 10.1093/database/baz046 30951143 10.1093/database/baz046 PMC6450036

[17] Frühwald MC, Hasselblatt M, Nemes K, Bens S, Steinbügl M, Johann PD et al (2020) Age and DNA methylation subgroup as potential independent risk factors for treatment stratification in children with atypical teratoid/rhabdoid tumors. Neuro Oncol 22:1006–1017. 10.1093/neuonc/noz244 31883020 10.1093/neuonc/noz244 PMC7339901

[18] Gastberger K, Fincke VE, Mucha M, Siebert R, Hasselblatt M, Frühwald MC (2023) Current molecular and clinical landscape of ATRT—the link to future therapies. Cancer Manag Res 15:1369–1393. 10.2147/CMAR.S379451 38089834 10.2147/CMAR.S379451 PMC10712249

[19] Gaujoux R, Seoighe C (2010) A flexible R package for nonnegative matrix factorization. BMC Bioinform 11:367. 10.1186/1471-2105-11-367 10.1186/1471-2105-11-367 PMC2912887 20598126

[20] Ge SX, Jung D, Yao R (2020) ShinyGO: a graphical gene-set enrichment tool for animals and plants. Bioinformatics 36:2628–2629. 10.1093/bioinformatics/btz931 31882993 10.1093/bioinformatics/btz931 PMC7178415

[21] Hao Y, Hao S, Andersen-Nissen E, Mauck WM, Zheng S, Butler A et al (2021) Integrated analysis of multimodal single-cell data. Cell 184:3573–3587.e29. 10.1016/j.cell.2021.04.048 34062119 10.1016/j.cell.2021.04.048 PMC8238499

[22] Hao Y, Stuart T, Kowalski MH, Choudhary S, Hoffman P, Hartman A et al (2024) Dictionary learning for integrative, multimodal and scalable single-cell analysis. Nat Biotechnol 42:293–304. 10.1038/s41587-023-01767-y 37231261 10.1038/s41587-023-01767-y PMC10928517

[23] Hasselblatt M, Nagel I, Oyen F, Bartelheim K, Russell RB, Schüller U et al (2014) *SMARCA4*-mutated atypical teratoid/rhabdoid tumors are associated with inherited germline alterations and poor prognosis. Acta Neuropathol 128:453–456. 10.1007/s00401-014-1323-x 25060813 10.1007/s00401-014-1323-x

[24] Heumos L, Schaar AC, Lance C, Litinetskaya A, Drost F, Zappia L et al (2023) Best practices for single-cell analysis across modalities. Nat Rev Genet 24:550–572. 10.1038/s41576-023-00586-w 37002403 10.1038/s41576-023-00586-w PMC10066026

[25] Holdhof D, Johann PD, Spohn M, Bockmayr M, Safaei S, Joshi P et al (2020) Atypical teratoid/rhabdoid tumors (ATRTs) with *SMARCA4* mutation are molecularly distinct from *SMARCB1*-deficient cases. Acta Neuropathol 141:291–301. 10.1007/s00401-020-02250-7 33331994 10.1007/s00401-020-02250-7 PMC7847432

[26] Hothorn T (2025) maxstat: Maximally Selected Rank Statistics. The Comprehensive R Archive Network

[27] Jiang H, Lei R, Ding S-W, Zhu S (2014) Skewer: a fast and accurate adapter trimmer for next-generation sequencing paired-end reads. BMC Bioinform 15:182. 10.1186/1471-2105-15-182 10.1186/1471-2105-15-182 PMC4074385 24925680

[28] Johann PD, Erkek S, Zapatka M, Kerl K, Buchhalter I, Hovestadt V et al (2016) Atypical teratoid/rhabdoid tumors are comprised of three epigenetic subgroups with distinct enhancer landscapes. Cancer Cell 29:379–393. 10.1016/j.ccell.2016.02.001 26923874 10.1016/j.ccell.2016.02.001

[29] Johann PD, Altendorf L, Efremova EM, Holsten T, Steinbügl M, Nemes K et al (2023) Recurrent atypical teratoid/rhabdoid tumors (AT/RT) reveal discrete features of progression on histology, epigenetics, copy number profiling, and transcriptomics. Acta Neuropathol 146:527–541. 10.1007/s00401-023-02608-7 37450044 10.1007/s00401-023-02608-7 PMC10412492

[30] Kaplan EL, Meier P (1958) Nonparametric estimation from incomplete observations. J Am Stat Assoc 53:457–481

[31] Kennedy N (2020) forestmodel: Forest Plots from Regression Models. The Comprehensive R Archive Network

[32] Kolberg L, Raudvere U, Kuzmin I, Adler P, Vilo J, Peterson H (2023) g:Profiler—interoperable web service for functional enrichment analysis and gene identifier mapping (2023 update). Nucleic Acids Res 51:W207–W212. 10.1093/nar/gkad347 37144459 10.1093/nar/gkad347 PMC10320099

[33] Kolde R (2025) pheatmap: Pretty Heatmaps. The Comprehensive R Archive Network

[34] Korsunsky I, Millard N, Fan J, Slowikowski K, Zhang F, Wei K et al (2019) Fast, sensitive and accurate integration of single-cell data with Harmony. Nat Methods 16:1289–1296. 10.1038/s41592-019-0619-0 31740819 10.1038/s41592-019-0619-0 PMC6884693

[35] Liu X, Zhu R, Luo Y, Wang S, Zhao Y, Qiu Z et al (2021) Distinct human Langerhans cell subsets orchestrate reciprocal functions and require different developmental regulation. Immunity 54:2305–2320.e11. 10.1016/j.immuni.2021.08.012 34508661 10.1016/j.immuni.2021.08.012

[36] Love MI, Huber W, Anders S (2014) Moderated estimation of fold change and dispersion for RNA-seq data with DESeq2. Genome Biol 15:1–21. 10.1186/s13059-014-0550-8 10.1186/s13059-014-0550-8 PMC4302049 25516281

[37] Monti S, Tamayo P, Mesirov J, Golub T (2003) Consensus clustering: a resampling-based method for class discovery and visualization of gene expression microarray data. Mach Learn 52:91–118. 10.1023/A:1023949509487

[38] Mulder K, Patel AA, Kong WT, Piot C, Halitzki E, Dunsmore G et al (2021) Cross-tissue single-cell landscape of human monocytes and macrophages in health and disease. Immunity 54:1883–1900.e5. 10.1016/j.immuni.2021.07.007 34331874 10.1016/j.immuni.2021.07.007

[39] Neftel C, Laffy J, Filbin MG, Hara T, Shore ME, Rahme GJ et al (2019) An integrative model of cellular states, plasticity, and genetics for glioblastoma. Cell 178:835–849.e21. 10.1016/j.cell.2019.06.024 31327527 10.1016/j.cell.2019.06.024 PMC6703186

[40] Newman AM, Liu CL, Green MR, Gentles AJ, Feng W, Xu Y et al (2015) Robust enumeration of cell subsets from tissue expression profiles. Nat Methods 12:453–457. 10.1038/nmeth.3337 25822800 10.1038/nmeth.3337 PMC4739640

[41] Pombo Antunes AR, Scheyltjens I, Lodi F, Messiaen J, Antoranz A, Duerinck J et al (2021) Single-cell profiling of myeloid cells in glioblastoma across species and disease stage reveals macrophage competition and specialization. Nat Neurosci 24:595–610. 10.1038/s41593-020-00789-y 33782623 10.1038/s41593-020-00789-y

[42] Povedano JM, Li V, Lake KE, Bai X, Rallabandi R, Kim J et al (2022) TK216 targets microtubules in Ewing sarcoma cells. Cell Chem Biol 29:1325–1332.e4. 10.1016/j.chembiol.2022.06.002 35803262 10.1016/j.chembiol.2022.06.002 PMC9394687

[43] R Core Team (2013) R: a language and environment for statistical computing. R Foundation for Statistical Computing. The Comprehensive R Archive Network

[44] Reimand J, Kull M, Peterson H, Hansen J, Vilo J (2007) g:Profiler—a web-based toolset for functional profiling of gene lists from large-scale experiments. Nucleic Acids Res 35:W193–W200. 10.1093/nar/gkm226 17478515 10.1093/nar/gkm226 PMC1933153

[45] Rorke LB, Packer R, Biegel J (1995) Central nervous system atypical teratoid/rhabdoid tumors of infancy and childhood. J Neurooncol 24:21–28. 10.1007/BF01052653 8523069 10.1007/BF01052653

[46] Rorke LB, Packer RJ, Biegel JA (1996) Central nervous system atypical teratoid/rhabdoid tumors of infancy and childhood: definition of an entity. J Neurosurg 85:56–65. 10.3171/jns.1996.85.1.0056 8683283 10.3171/jns.1996.85.1.0056

[47] Sacco JL, Gomez EW (2024) Epithelial–mesenchymal plasticity and epigenetic heterogeneity in cancer. Cancers (Basel). 10.3390/cancers16193289 39409910 10.3390/cancers16193289 PMC11475326

[48] Saitoh M (2018) Involvement of partial EMT in cancer progression. J Biochem 164:257–264. 10.1093/jb/mvy047 29726955 10.1093/jb/mvy047

[49] Sakamoto K, Endo K, Sakamoto K, Kayamori K, Ehata S, Ichikawa J et al (2021) EHF suppresses cancer progression by inhibiting ETS1-mediated ZEB expression. Oncogenesis. 10.1038/s41389-021-00313-2 33712555 10.1038/s41389-021-00313-2 PMC7955083

[50] Schneppenheim R, Frühwald MC, Gesk S, Hasselblatt M, Jeibmann A, Kordes U et al (2010) Germline nonsense mutation and somatic inactivation of SMARCA4/BRG1 in a family with rhabdoid tumor predisposition syndrome. Am J Hum Genet 86:279–284. 10.1016/j.ajhg.2010.01.013 20137775 10.1016/j.ajhg.2010.01.013 PMC2820190

[51] Sharma R, Zhang C, Narendran A (2023) The small-molecule E26-transformation-specific inhibitor TK216 attenuates the oncogenic properties of pediatric leukemia. Genes (Basel) 14:1916. 10.3390/genes14101916 37895265 10.3390/genes14101916 PMC10606408

[52] Sjoberg D, Baillie M, Fruechtenicht C, Haesendonckx S, Treis T (2025) ggsurvfit: Flexible Time-to-Event Figures. The Comprehensive R Archive Network

[53] Spriano F, Chung EYL, Gaudio E, Tarantelli C, Cascione L, Napoli S et al (2019) The ETS inhibitors YK-4-279 and TK-216 are novel antilymphoma agents. Clin Cancer Res 25:5167–5176. 10.1158/1078-0432.CCR-18-2718 31182435 10.1158/1078-0432.CCR-18-2718

[54] Sun PP, Liao SX, Sang P, Liu MM, Yang JB (2024) Lysosomal transmembrane protein 5: impact on immune cell function and implications for immune-related deficiencies. Heliyon. 10.1016/j.heliyon.2024.e36705 39281638 10.1016/j.heliyon.2024.e36705 PMC11401081

[55] Therneau TM, Lumley T, Atkinson E, Crowson C (2024) A package for survival analysis in R. Comprehensive R Archive Network

[56] Tickle T, Tirosh I, Georgescu C, Brown M, Haas B (2019) inferCNV of the Trinity CTAT Project. Klarman Cell Observatory

[57] Van Lent J, Baggiolini A (2024) Harmony in chaos: understanding cancer through the lenses of developmental biology. Mol Oncol 18:793–796. 10.1002/1878-0261.13594 38282579 10.1002/1878-0261.13594 PMC10994237

[58] Wickham H (2016) ggplot2: elegant graphics for data analysis. Springer Nature

[59] Wilkerson MD, Hayes DN (2010) ConsensusClusterPlus: a class discovery tool with confidence assessments and item tracking. Bioinformatics 26:1572–1573. 10.1093/bioinformatics/btq170 20427518 10.1093/bioinformatics/btq170 PMC2881355

[60] Wilson BG, Roberts CWM (2011) SWI/SNF nucleosome remodellers and cancer. Nat Rev Cancer 11:481–492. 10.1038/nrc3068 21654818 10.1038/nrc3068

[61] Winkler J, Abisoye-Ogunniyan A, Metcalf KJ, Werb Z (2020) Concepts of extracellular matrix remodelling in tumour progression and metastasis. Nat Commun. 10.1038/s41467-020-18794-x 33037194 10.1038/s41467-020-18794-x PMC7547708

[62] Yasmin N, Konradi S, Eisenwort G, Schichl YM, Seyerl M, Bauer T et al (2013) β-catenin promotes the differentiation of epidermal Langerhans dendritic cells. J Investig Dermatol 133:1250–1259. 10.1038/jid.2012.481 23303458 10.1038/jid.2012.481

[63] Yi M, Li T, Niu M, Zhang H, Wu Y, Wu K et al (2024) Targeting cytokine and chemokine signaling pathways for cancer therapy. Signal Transduct Target Ther. 10.1038/s41392-024-01868-3 39034318 10.1038/s41392-024-01868-3 PMC11275440

[64] Yu G, Gao C-H (2025) enrichplot: Visualization of Functional Enrichment Result. The Comprehensive R Archive Network

[65] Yu G, He QY (2016) ReactomePA: an R/Bioconductor package for reactome pathway analysis and visualization. Mol Biosyst 12:477–479. 10.1039/c5mb00663e 26661513 10.1039/c5mb00663e

[66] Zin F, Cotter JA, Haberler C, Dottermusch M, Neumann J, Schüller U et al (2021) Histopathological patterns in atypical teratoid/rhabdoid tumors are related to molecular subgroup. Brain Pathol. 10.1111/bpa.12967 33938067 10.1111/bpa.12967 PMC8412123

