# Supplementary material for: Molecular changes during AT/RT progression associated with epithelial–mesenchymal transition and extracellular matrix changes
Source: Acta Neuropathol. 2026 Jul 1;152(1):1. doi: 10.1007/s00401-026-03050-1 (PMC13323348; doi:10.1007/s00401-026-03050-1)
Supplement: Supplementary file 1 — Supplementary file1 (PDF 83018 KB) [file 401_2026_3050_MOESM1_ESM.pdf]

Title

Molecular changes during AT/RT progression associated with epithelial-mesenchymal transition and extracellular matrix changes

Journal

Acta Neuropathologica

Authors

Lea Altendorf[1,2], Anton Althammer[1,2,3], Rajanya Roy[4], Karoline Hack[1,2], Flavia W. de Faria[4], Arend Koch[5], Vanessa Thaden[1,2], Melanie Schoof[1,2], Martin U. Schuhmann[6], Peter Hauser[7], Pascal D. Johann[8,9,10], Martin Hasselblatt[11], Michael C. Frühwald[8], Kornelius Kerl[4], and Ulrich Schüller*[1,2,12]

[1]Department of Pediatric Hematology and Oncology, University Medical Center Hamburg-Eppendorf, 20251 Hamburg, Germany

[2]Research Institute Children's Cancer, University Medical Center Hamburg-Eppendorf, 20251 Hamburg, Germany

[3]Mildred Scheel Cancer Career Center HaTriCS4, University Medical Center Hamburg-Eppendorf, 20251 Hamburg, Germany.

[4]Department of Pediatric Hematology and Oncology, University Medical Center Münster, 48149 Münster, Germany

[5]Institute of Neuropathology, Charité – Universitätsmedizin Berlin, 10117 Berlin, Germany

[6]Division of Pediatric Neurosurgery, Department of Neurosurgery, Eberhard Karl's University Hospital of Tübingen, 72076 Tübingen, Germany

[7]Second Department of Pediatrics, Semmelweis University, 1085 Budapest, Hungary

[8]Paediatric and Adolescent Medicine, Swabian Children's Cancer Center Augsburg, EU-RHAB trial center, 86156 Augsburg, Germany

[9]Hopp Children's Cancer Center (KiTZ), German Cancer Research Center (DKFZ) and German Cancer Research Consortium (DKTK), 69120 Heidelberg, Germany

[10]Division of Pediatric Neurooncology, German Cancer Research Center (DKFZ) and German Cancer Research Consortium (DKTK), 69120 Heidelberg, Germany

[11]Institute of Neuropathology, University Hospital Münster, 48149 Münster, Germany

[12]Institute of Neuropathology, University Medical Center Hamburg-Eppendorf, 20251 Hamburg, Germany

*Corresponding Author

Corresponding author

Ulrich Schüller, MD

Research Institute Children‘s Cancer Center

Martinistraße 52, N63, D-20251 Hamburg, Germany

Phone: +49-40-426051240

Fax: +49-40-741040350

E-mail: u.schueller@uke.de

Altendorf et al. Fig. S1

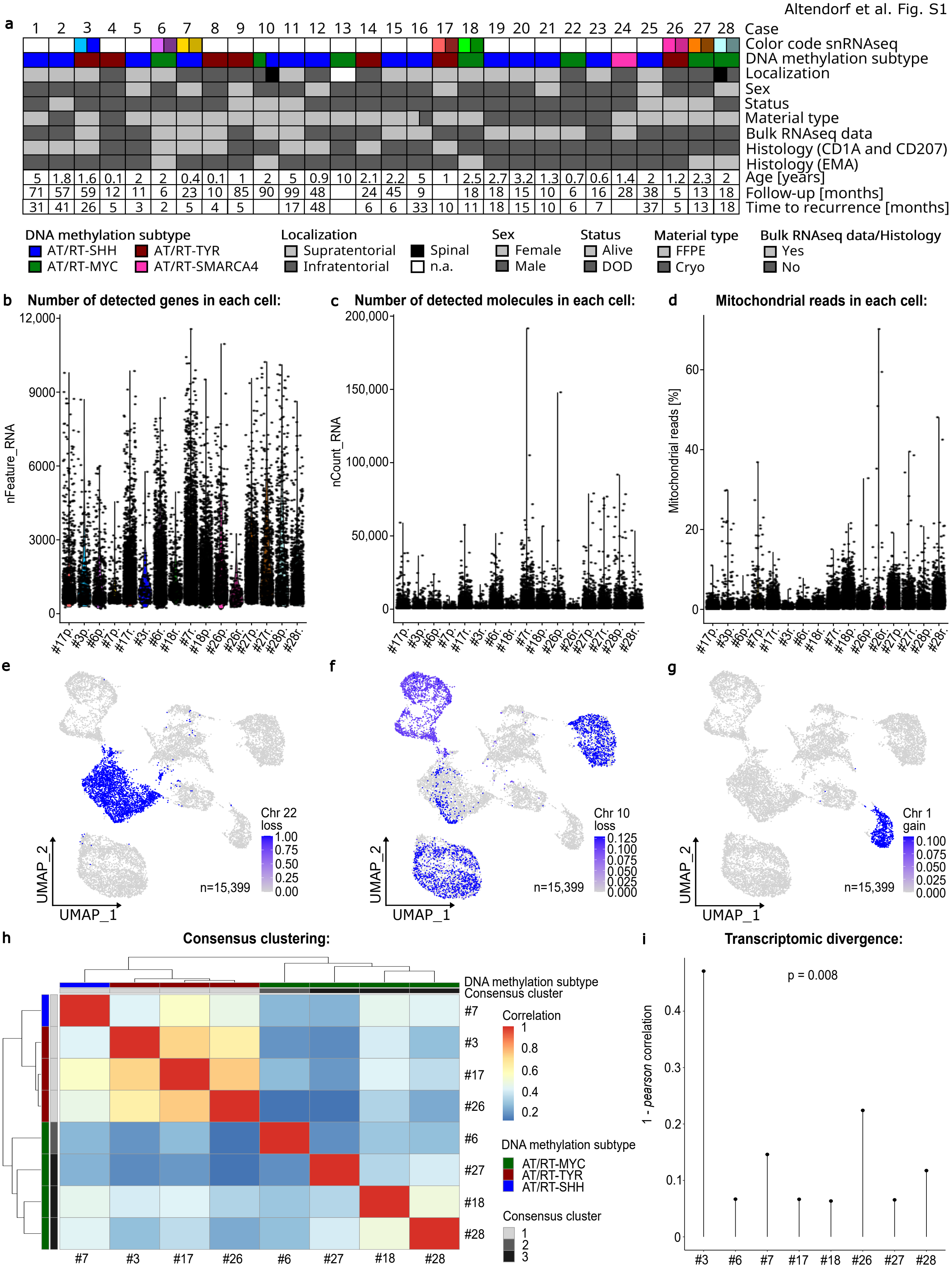

Fig. S1 SnRNAseq quality control and comparison of the gene expression profiles of all AT/RT primary-recurrence pairs including the TME. Overview of all samples and present data used in this study as extension to Johann, Altendorf et al. [26]. Case numbers 1-26 were assigned to the patients according to Johann, Altendorf et al. [26]. Cases 27 and 28 were added to this study (**a**). Violin plot showing the number of detected genes in each cell for each sample (**b**). Violin plot showing the number of detected molecules in each cell for each sample (**c**). Violin plot showing the proportion of reads matching mitochondrial genes (**d**). UMAP of all cells (n = 15,399) annotated by chromosome 22 status (**e**). UMAP of all cells (n = 15,399) annotated by chromosome 10 status (**f**). UMAP of all cells (n = 15,399) annotated by chromosome 1 status (**g**). Consensus clustering of pseudobulk values for each patient. The 1000 most variable genes were chosen and the optimal k was calculated as k = 3 (**h**). Lollipop plot showing the transcriptomic divergence between each primary tumor sample and paired recurrence. Significant different gene expression profiles were observed for each pair (p = 0.008, two-sided Wilcoxon test) (**i**).

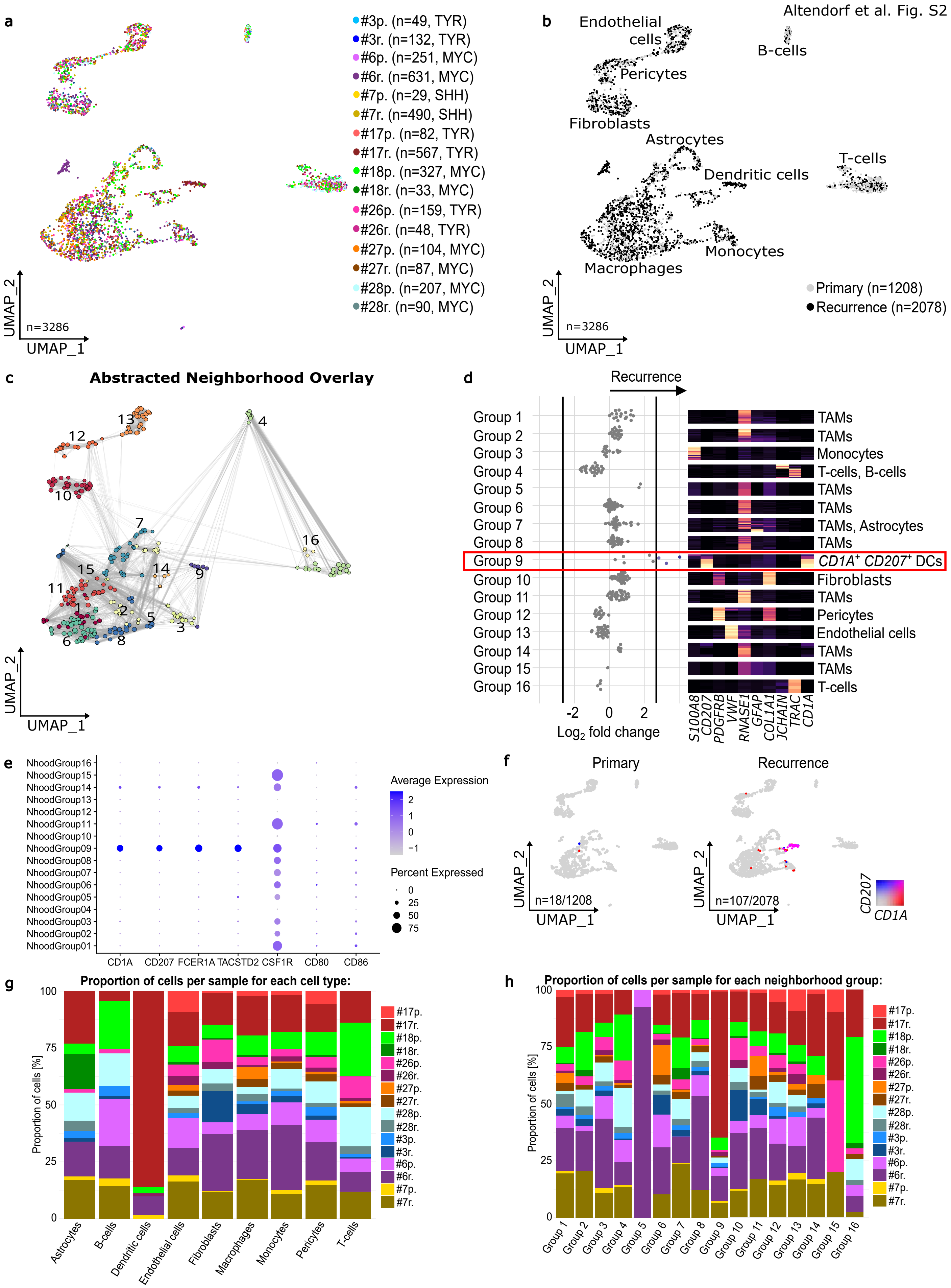

Fig. S2 Annotation and investigation of cellular abundances of the TME. UMAPs using 10 neighboring points of harmony-integrated data of the TME only (n = 3286), annotated by samples (**a**) and status (primary or recurrence) and cell types (**b**). Assignment of AT/RT TME cells to partially overlapping neighborhoods on the UMAP of A and B using *miloR* (**c**). GLM comparing primary versus recurrent AT/RT TME cells. Each dot represents one neighborhood (**d**). Dot plot showing differential gene expression analysis of neighborhood group 9 vs. all other neighborhood groups (**e**). UMAPs of the TME only (n = 3286), annotated by *CD1A*$^{+}$ *CD207*$^{+}$ expression in primary (left, n = 18/1208 positive cells) and recurrent TME cells (right, n = 107/2078 positive cells) (**f**). Barplots showing the proportion of cells of each sample for each TME cell type (**g**) and for each neighborhood group (**h**).

Altendorf et al. Fig. S3

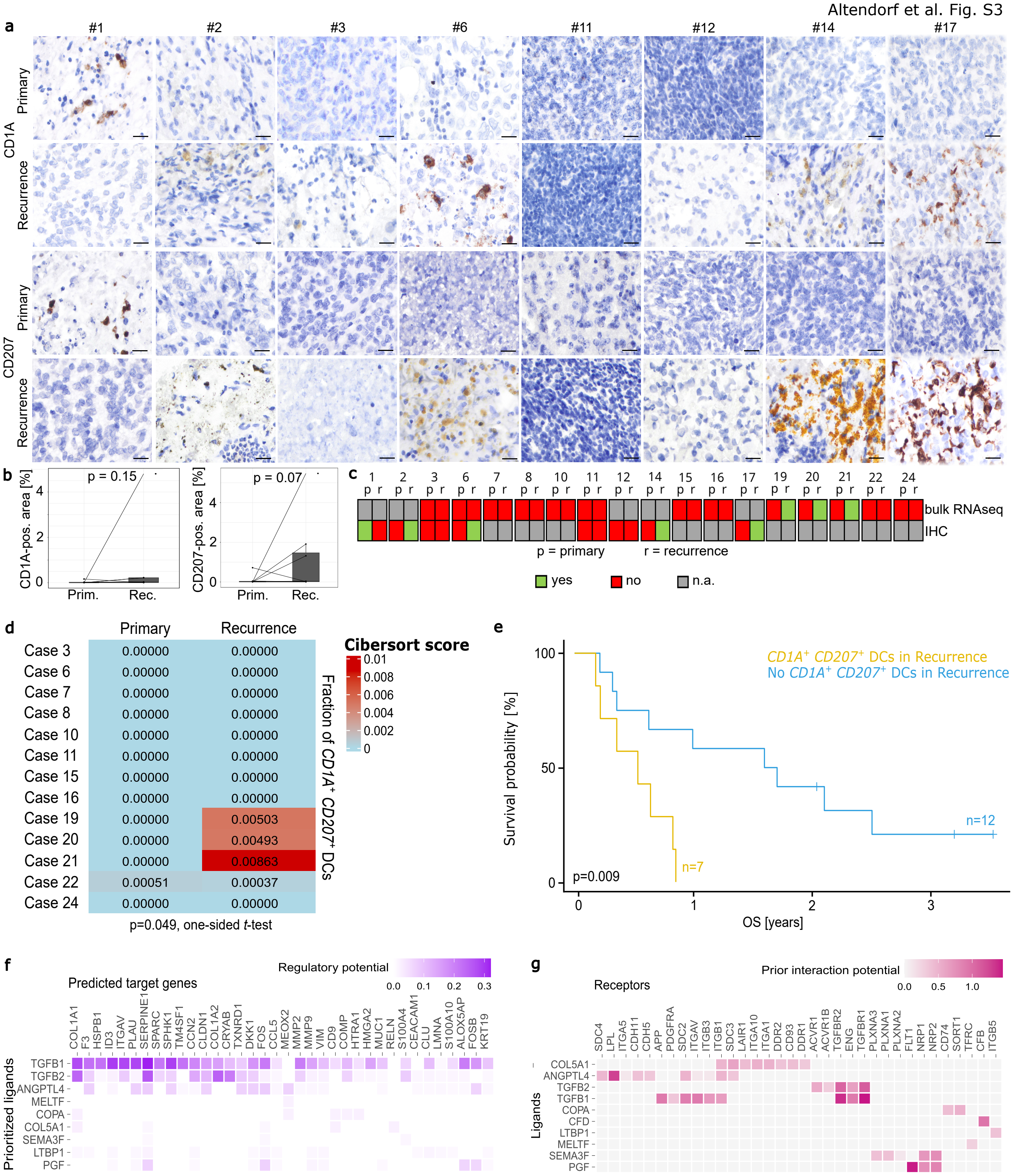

Fig. S3 Validation and characterization of CD1A$^+$ and CD207$^+$ DCs in AT/RT recurrences. CD1A/CD207 IHC of AT/RT primary-recurrence pairs. Scale bars indicate 20 µm (**a**). Boxplots showing IHC quantification of the CD1A-/CD207-positive area [%] of each primary tumor sample and paired recurrence (**b**). Correlation of CD1A/CD207 expression observed in IHC and bulk RNAseq data (**c**). Heatmap of *CIBERSORT* results for the deconvolution of previously published bulk RNAseq data of paired primary and recurrent AT/RT [26] revealed enrichment of *CD1A*$^+$ *CD207*$^+$ DCs in recurrent AT/RT (p = 0.049, one-sided *t*-test) (**d**). OS of a cohort of AT/RT patients separated into two groups based on the abundance of *CD1A*$^+$ *CD207*$^+$ cells in the respective recurrent tumor samples. Patients with *CD1A*$^+$ *CD207*$^+$ DCs had a significantly shorter OS compared to patients without (p = 0.009, two-sided Log-Rank test) (**e**). Cell-cell communication results showing ligand-target gene interactions (**f**). Cell-cell communication results showing ligand-receptor interactions (**g**).

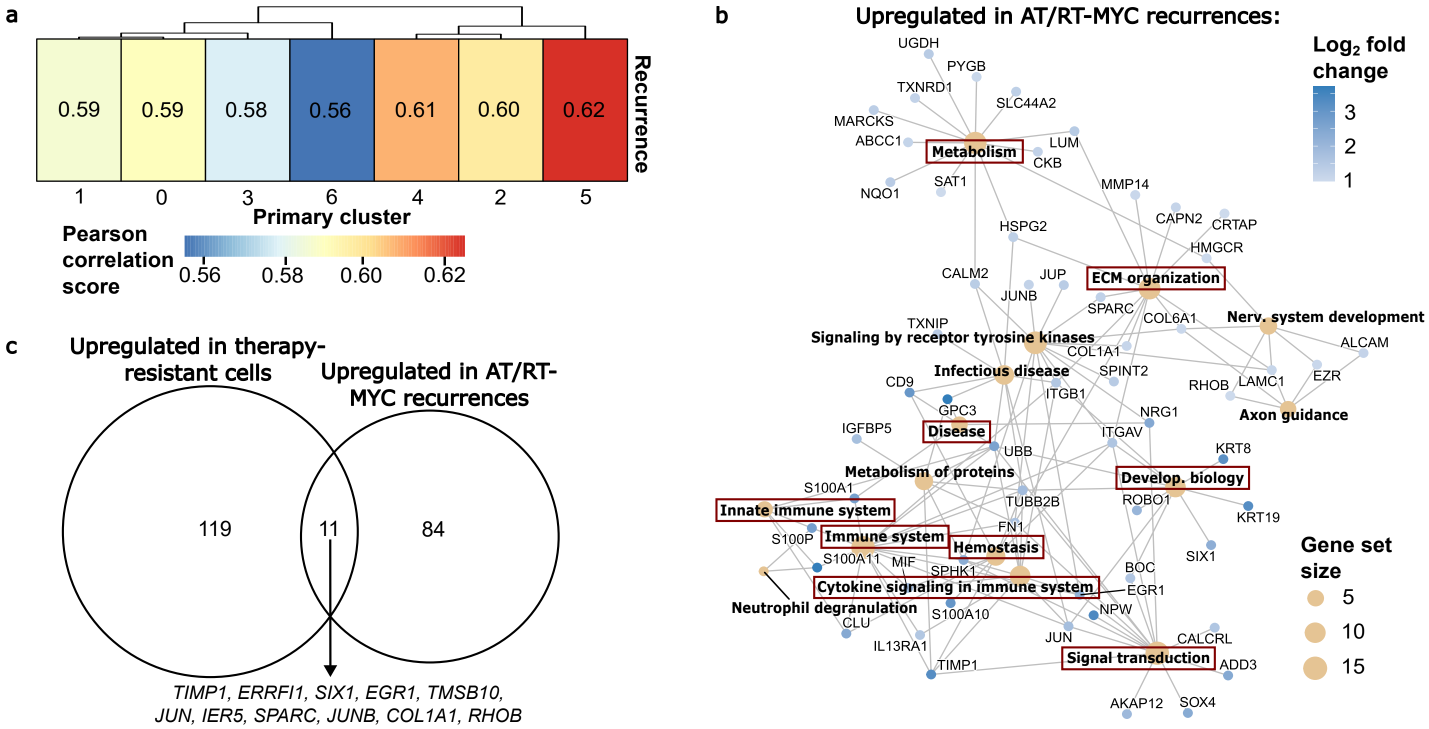

Fig. S4 Differentially expressed genes and pathways between AT/RT-MYC primary tumors and recurrences. Pearson correlation indicating highest similarity of AT/RT-MYC primary tumor cluster 2, 4, and 5 to recurrences (**a**). Upregulated genes and *Reactome* pathways in AT/RT-MYC recurrence tumor cells compared to primary tumor cells. The pathways marked in red were overlapping with upregulated pathways in potentially therapy resistant cells (primary tumor cluster 2, 4, and 5) (**b**). Venn diagram showing overlap of genes upregulated in potentially therapy-resistant cells (primary tumor cluster 2, 4, and 5) and AT/RT-MYC recurrences (**c**).

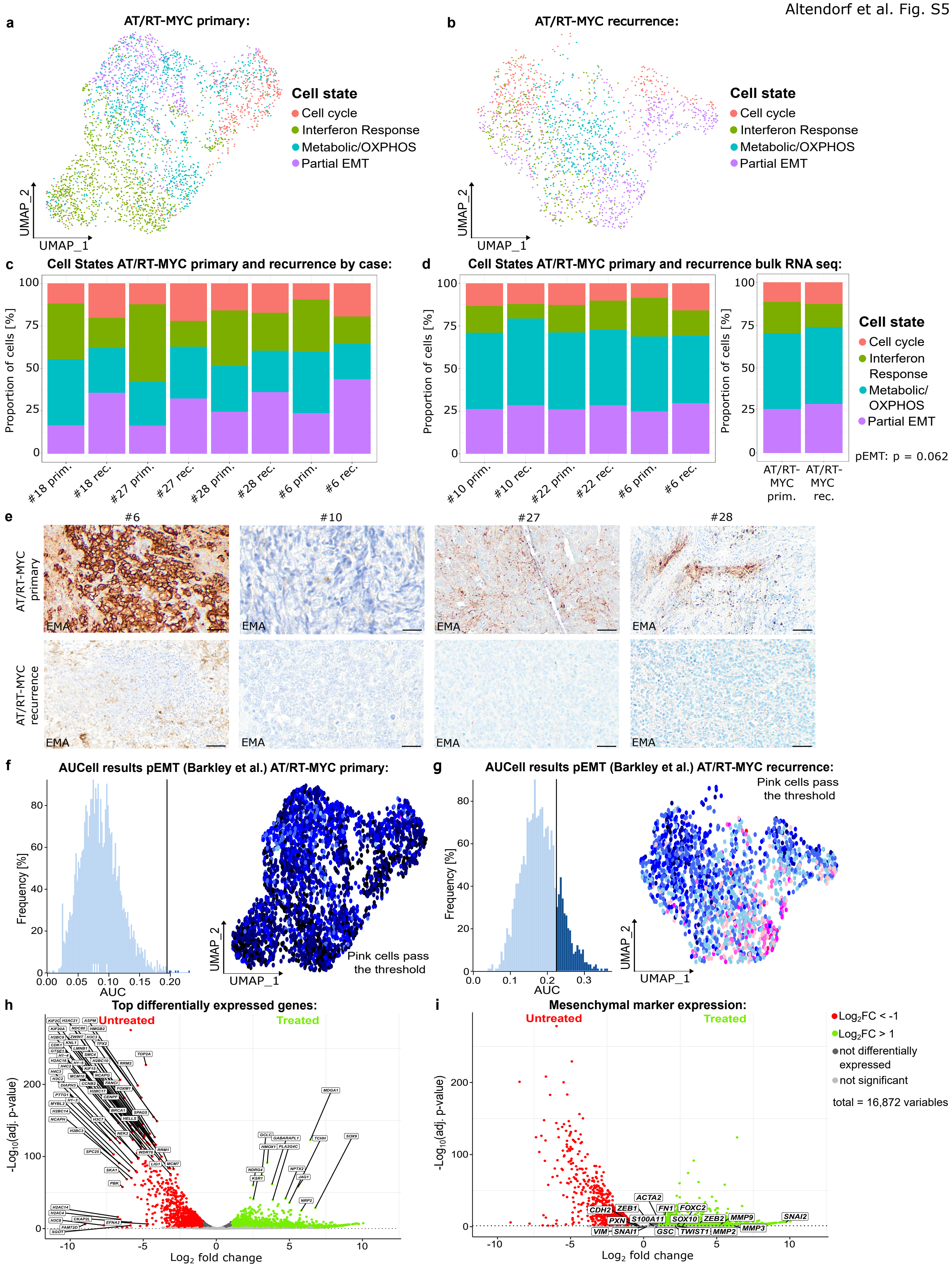

Fig. S5 Cancer cell states of AT/RT-MYC snRNAseq and bulk RNAseq data and bulk RNAseq data of AT/RT-MYC cell lines. Integrated UMAPs using 10 neighboring points of all AT/RT-MYC primary (n = 3188) (**a**) and recurrence tumor cells (n = 1859) (**b**), annotated by cancer cell states resulting from NMF analysis. Distribution of cancer cell states of AT/RT-MYC recurrence compared to primary tumor cells for each case (**c**). Distribution of cancer cell states using bulk RNA sequencing data of each AT/RT-MYC case and of AT/RT-MYC recurrences compared to primary tumors (pEMT: $p = 0.062$, two-sided paired *t*-test) (**d**). EMA IHC of AT/RT primary-recurrence pairs. Scale bars indicate 50 µm (**e**). *AUCell* results of AT/RT-MYC primary (**f**) and recurrence tumor cells (**g**) undergoing pEMT (according to Barkley et al. [5]). The histograms were used to define the AUC thresholds (0.195 and 0.225). All cells that passed the threshold were coloured in pink in the UMAPs. Volcano Plots of bulk RNAseq data showing differentially expressed genes of BT-16 and CHLA-266 untreated (red) vs. BT-16 and CHLA-266 treated (green) with the top differentially expressed genes (**h**) or mesenchymal markes (**i**) annotated.

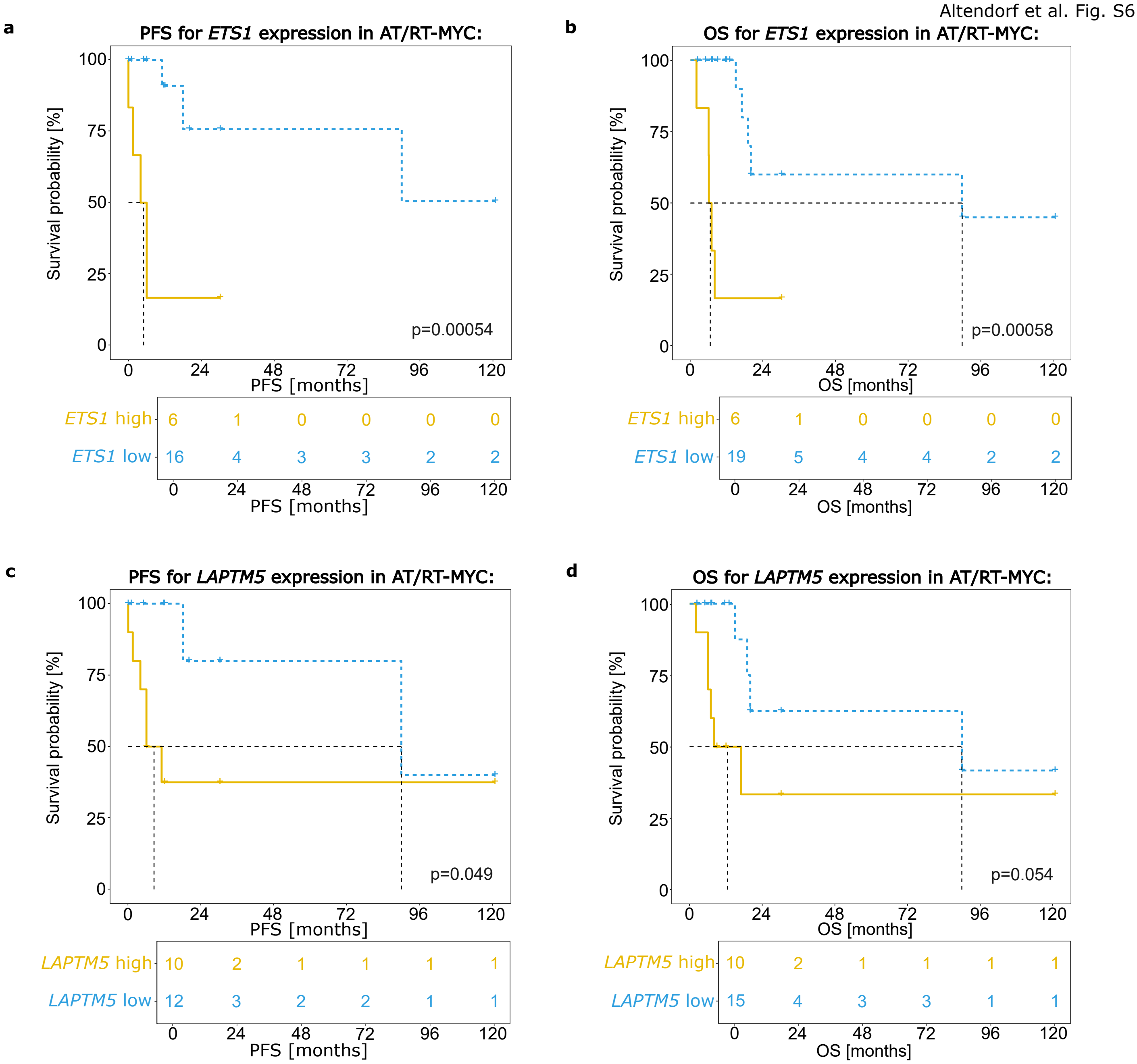

Fig. S6 Survival analyses of AT/RT-MYC patients in correlation with the expression of the top upregulated genes identified in potentially therapy-resistant primary tumor cells. PFS of a cohort of AT/RT-MYC patients separated into two groups using the best cutoff for *ETS1* expression. The group with high *ETS1* expression (n = 6) had a significantly worse PFS compared to the group with low *ETS1* expression (n = 16, p = 0.00054, two-sided Log-Rank test) (**a**). OS of a cohort of AT/RT-MYC patients separated into two groups using the best cutoff for *ETS1* expression. The group with high *ETS1* expression (n = 6) had a significantly worse OS compared to the group with low *ETS1* expression (n = 19, p = 0.00058, two-sided Log-Rank test) (**b**). PFS of a cohort of AT/RT-MYC patients separated into two groups using the best cutoff for *LAPTM5* expression. The group with high *LAPTM5* expression (n = 10) had a significantly worse OS compared to the group with low *LAPTM5* expression (n = 12, p = 0.049, two-sided Log-Rank test) (**c**). OS of a cohort of AT/RT-MYC patients separated into two groups using the best cutoff for *LAPTM5* expression. The group with high *LAPTM5* expression (n = 10) had no significantly different OS compared to the group with low *LAPTM5* expression (n = 15, p = 0.054, two-sided Log-Rank test) (**d**).
